# Supplementary material for: Protective Effects of Marine Alkaloid Neolamellarin A Derivatives against Glutamate Induced PC12 Cell Apoptosis
Source: Mar Drugs. 2022 Apr 12;20(4):262. doi: 10.3390/md20040262 (PMC9026748; doi:10.3390/md20040262)
Supplement: Supplementary file 1 [file marinedrugs-20-00262-s001.zip › marinedrugs-1668363-Supplementary Materials.pdf]

Supplementary Material

Protective Effects of Marine alkaloid Neolamellarin A Derivatives against Glutamate Induced PC12 Cell Apoptosis

Kai Zhang [1,†], Xian Guan [1,†], Xiao Zhang [1], Lu Liu [3], Ruijuan Yin [1,3,*], and Tao Jiang [1,2,*]

1. Key Laboratory of Marine Drugs, Ministry of Education, School of Medicine and Pharmacy, Ocean University of China, Qingdao 266003, P. R. China; zk15065683854@163.com(K.Z.); 17806022265@163.com(X.Z.); 18838966959@163.com (X.G.)

2. Laboratory for Marine Drugs and Bioproducts of Qingdao National Laboratory for Marine Science and Technology, Qingdao 266237, P. R. China;

3. Marine Biomedical Research Institute of Qiangdao, Qingdao 266237, P. R. China；ll3049@yeah.net (L.L.)

*Correspondence: yinruijuan@ouc.edu.cn (R.Y.); jiangtao@ouc.edu.cn (T.J.);

Tel.: +86-532-8590-6853 (R.Y.)

#These authors contributed equally to this work.

Contents

General procedures for the synthesis of important intermediates 1

Figure S1. HRMS spectrum of **4a** 3

Figure S2. ^{1}H NMR spectrum of **4a** 3

Figure S3. ^{13}C NMR spectrum of **4a** 4

Figure S4. HRMS spectrum of **4b** 4

Figure S5. ^{1}H NMR spectrum of **4b** 5

Figure S6. ^{13}C NMR spectrum of **4b** 5

Figure S7. ^{1}H NMR spectrum of **4c** 6

Figure S8. ^{13}C NMR spectrum of **4c** 6

Figure S9. HRMS spectrum of **4d** 7

Figure S10. ^{1}H NMR spectrum of **4d** 7

Figure S11. ^{13}C NMR spectrum of **4d** 8

Figure S12. HRMS spectrum of **4e** 8

Figure S13. ^{1}H NMR spectrum of **4e** 9

Figure S14. ^{13}C NMR spectrum of **4e** 9

Figure S15. HRMS spectrum of **5a** 10

Figure S16. ^{1}H NMR spectrum of **5a** 10

Figure S17. ^{13}C NMR spectrum of **5a** 11

Figure S18. HRMS spectrum of **5b** 11

Figure S19. ^{1}H NMR spectrum of **5b** 12

Figure S20. ^{13}C NMR spectrum of **5b** 12

Figure S21. HRMS spectrum of **5c** 13

Figure S22. ^{1}H NMR spectrum of **5c** 13

Figure S23. ^{13}C NMR spectrum of **5c** 14

Figure S24. HRMS spectrum of **5d** 14

Figure S25. ^{1}H NMR spectrum of **5d** 15

Figure S26. ^{13}C NMR spectrum of **5d** 15

Figure 27. HRMS spectrum of **5e** 16

Figure S28. ^{1}H NMR spectrum of **5e** 16

Figure S29. ^{13}C NMR spectrum of **5e** 17

Figure S30. HRMS spectrum of **1a** 17

Figure S31. ^{1}H NMR spectrum of **1a** 18

Figure S32. ^{13}C NMR spectrum of **1a** 18

Figure S33. HRMS spectrum of **1b** 19

Figure S34. ^{1}H NMR spectrum of **1b** 19

Figure S35. ^{13}C NMR spectrum of **1b** 20

Figure S36. HRMS spectrum of **1c** 20

Figure S37. ^{1}H NMR spectrum of **1c** 21

Figure S38. ^{13}C NMR spectrum of **1c** 21

Figure S39. HRMS spectrum of **1d** 22

Figure S40. ^{1}H NMR spectrum of **1d** 22

Figure S41. ^{13}C NMR spectrum of **1d** 23

Figure S42. HRMS spectrum of **1e** 23

Figure S43. ^{1}H NMR spectrum of **1e** 24

Figure S44. ^{13}C NMR spectrum of **1e** 24

Figure S45. HRMS spectrum of **1f** 25

Figure S46. ^{1}H NMR spectrum of **1f** 25

Figure S47. ^{13}C NMR spectrum of **1f** 26

Figure S48. HRMS spectrum of **1g** 26

Figure S49. ^{1}H NMR spectrum of **1g** 27

Figure S50. ^{13}C NMR spectrum of **1g** 27

Figure S51. HRMS spectrum of **1h** 28

Figure S52. ^{1}H NMR spectrum of **1h** 28

Figure S53. ^{13}C NMR spectrum of **1h** 29

Figure S54. HRMS spectrum of **1i** 29

Figure S55. ^{1}H NMR spectrum of **1i** 30

Figure S56. ^{13}C NMR spectrum of **1i** 30

Figure S57. HRMS spectrum of **1j** 31

Figure S58. ^{1}H NMR spectrum of **1j** 31

Figure S59. ^{13}C NMR spectrum of **1j** 32

Figure S60. HRMS spectrum of **1k** 32

Figure S61. ^{1}H NMR spectrum of **1k** 33

Figure S62. ^{13}C NMR spectrum of **1k** 33

Figure S63. HRMS spectrum of **1l** 34

Figure S64. ^{1}H NMR spectrum of **1l** 34

Figure S65. ^{13}C NMR spectrum of **1l** 35

Figure S66. HRMS spectrum of **1m** 35

Figure S67. ^{1}H NMR spectrum of **1m** 36

Figure S68. ^{13}C NMR spectrum of **1m** 36

Figure S69. ^{1}H NMR spectrum of **1n** 37

Figure S70. ^{13}C NMR spectrum of **1n** 37

Figure S71. HRMS spectrum of **1o** 38

Figure S72. ^{1}H NMR spectrum of **1o** 38

Figure S73. ^{13}C NMR spectrum of **1o** 39

Table S1 The cytotoxicity of the permethylated Neolamellarin A derivatives **1a - 1o** at gradient concentrations on PC12 cell line 40

Table S2 The neuroprotective activity of the permethylated Neolamellarin A derivatives **1a - 1o** at gradient concentrations on PC12 cell death induced by glutamate 42

General procedures for the synthesis of important intermediates

The general procedures for the synthesis of important intermediates **4a - 4e**, **5a - 5e** and **6a - 6c** are as follows.

1.1. General procedure for the synthesis of **4a - 4e**

The substituted phenyl acetaldehydes **3** [17] (10 mmol) and benzylamine (1.07 g, 10 mmol) were dissolved in THF (15 mL), and stirred at room temperature for 0.5 h under argon. Then AgOAc (3.34 g, 20 mmol) and NaOAc (1.64 g, 20 mmol) were added, and the mixture was stirred at 60 °C for 8 h. The mixture was cooled down to room temperature, and then the resulting precipitate was filtered and washed with EtOAc. The filtrate was evaporated under reduced pressure and the resulting residue was purified by flash column chromatography (petroleum ether: dichloromethane = 5: 1 to 3: 1) to give the pure compounds **4a - 4e**.

1.1.1. 1-benzyl-3,4-bis(2-methoxyphenyl)-1*H*-pyrrole (**4a**)

White solid 1.15 g. Yield 62.3%. ^{1}H NMR (500 MHz, $CDCl_3$) δ (ppm) 7.37-7.36 (m, 2H), 7.31-7.26 (m, 3H), 7.14-7.13 (m, 4H), 6.91 (s, 2H), 6.82-6.81 (m, 4H), 5.12 (s, 2H), 3.49 (s, 6H). ^{13}C NMR (125 MHz, $CDCl_3$) δ (ppm) 156.28 (2C), 137.53 (C), 130.44 (2CH), 128.65 (2CH), 127.67 (4CH), 126.60 (2CH), 126.05 (2CH), 121.19 (2C), 120.13 (CH), 119.97 (2C), 110.68 (2CH), 54.96 (CH_2), 53.54 ($2CH_3$). HRMS (ESI) m/z: calcd for M^+ $C_{25}H_{24}O_2N$, 370.1802; found, M^+ 370.1802.

1.1.2. 1-benzyl-3,4-bis(3-methoxyphenyl)-1*H*-pyrrole (**4b**)

White solid 1.04 g. Yield 56.5%. ^{1}H NMR (500 MHz, $CDCl_3$) δ (ppm) 7.37-7.35 (m, 2H), 7.33-7.32 (m, 1H), 7.26-7.25 (m, 2H), 7.17-7.14 (dd, J = 9.6, 6.2 Hz, 2H), 6.88-6.87 (d, J = 7.3 Hz, 2H), 6.83- 6.81(m, 4H), 6.74-6.72 (dd, J = 7.3, 2.3 Hz, 2H), 5.09 (s, 2H), 3.68 (s, 6H). ^{13}C NMR (125 MHz, $CDCl_3$) δ (ppm) 159.27 (2C), 137.26 (C), 137.07 (2C), 129.00 (2CH), 128.80 (2CH), 127.90 (CH), 127.42 (2CH), 123.45 (2C), 120.95 (2CH), 120.70 (2CH), 113.57 (2CH), 111.55 (2CH), 55.02 (CH_2), 53.58 ($2CH_3$). HRMS (ESI) m/z: calcd for M^+ $C_{25}H_{24}O_2N$, 370.1802; found, M^+ 370.1799.

1.1.3. 1-benzyl-3,4-bis(4-methoxyphenyl)-1*H*-pyrrole (**4c**)

White solid 1.34 g. Yield 72.8%. ^{1}H NMR (600 MHz, $CDCl_3$) δ (ppm) 7.38-7.36 (td, J = 7.4, 1.4 Hz, 2H), 7.33-7.31 (m, 1H), 7.27 –7.25 (m, 2H), 7.20-7.19 (d, J = 9.44 Hz, 4H), 6.82-6.80 (d, J = 9.44 Hz, 4H), 6.74 (d, J = 1.6 Hz, 2H), 5.07 (s, 2H), 3.80 (s, 6H). ^{13}C NMR (150 MHz, $CDCl_3$) δ (ppm) 157.66 (2C), 137.52 (C), 129.40 (4CH), 128.75 (2CH), 128.44 (CH), 127.80 (2C), 127.40 (2CH), 123.00 (2C), 120.02 (2CH), 113.56 (4CH), 55.15 ($2CH_3$), 53.50 (CH_2). HRMS (ESI) m/z: calcd for M^+ $C_{25}H_{24}O_2N$, 370.1802; found, M^+ 370.1798.

1.1.4 1-benzyl-3,4-bis(3,4-dimethoxyphenyl)-1*H*-pyrrole (**4d**)

White solid 1.41 g. Yield 65.4%. ^{1}H NMR (500 MHz, $CDCl_3$) δ (ppm) 7.38-7.35 (t, J = 7.0 Hz, 2H), 7.32-7.31 (d, J = 6.3 Hz, 1H), 7.25 (s, 2H), 6.84-6.82 (d, J = 8.1 Hz, 2H), 6.78-6.77 (m, 6H), 5.07 (s, 2H), 3.85 (s, 6H), 3.67 (s, 6H). ^{13}C NMR (125 MHz, $CDCl_3$) δ (ppm) 148.28 (2C), 147.02 (2C), 137.41 (C), 128.77 (2CH), 128.59 (2C), 127.84 (CH), 127.36 (2CH), 123.16 (2C), 120.39 (2CH), 119.99 (2CH), 111.99 (2CH), 110.93 (2CH), 55.80 ($2CH_3$), 55.57 ($2CH_3$), 53.53 (CH_2). HRMS (ESI) m/z: calcd for M^+ $C_{27}H_{28}O_4N$, 430.2013; found, M^+ 430.2011.

1.1.5 1-benzyl-3,4-bis(3,4,5-trimethoxyphenyl)-1*H*-pyrrole (**4e**)

White solid 1.98 g. Yield 73.8%. ^{1}H NMR (500 MHz, $CDCl_3$) δ (ppm) 7.38-7.36 (dd, J = 8.9, 4.0 Hz, 2H), 7.33-7.32 (d, J = 6.1 Hz, 1H), 7.26-7.25 (d, J = 6.1 Hz, 2H), 6.82 (s, 2H), 6.49 (s, 4H), 5.09 (s, 2H), 3.81 (s, 6H), 3.69 (s, 12H). ^{13}C NMR (125 MHz, $CDCl_3$) δ(ppm) 152.72 (4C), 137.25 (2C), 136.03 (C), 131.19 (2C), 128.81 (2CH), 127.92 (CH), 127.31 (2CH), 123.48 (2C), 120.19 (2CH), 105.55 (4CH), 60.87 ($2CH_3$), 55.86 ($4CH_3$), 53.58 (CH_2). HRMS (ESI) m/z: calcd for M^+ $C_{29}H_{32}O_6N$, 490.2224; found, M^+ 490.2214.

1.2. General procedure for the synthesis of **5a - 5e**

To a mixture of **4a - 4e** (2.0 mmol) and *t*-BuOK (22.24 g, 20.0 mmol) was successively added THF (12 mL) and DMSO (20 mL) under oxygen atmosphere, then the solution was stirred at room temperature for 1 h. The mixture was quenched with saturated aqueous NH_4Cl solution (30 mL) and extracted with EtOAc (20 × 3 mL). The organic phase was washed with H_2O (3 × 30 mL), dried with $MgSO_4$, and evaporated under reduced pressure and the resulting residue was purified by flash column chromatography (petroleum ether: ethyl acetate = 1: 1) to give the pure compounds **5a - 5e**.

1.2.1. 3,4-bis(2-methoxyphenyl)-1*H*-pyrrole (**5a**)

White solid 0.49 g. Yield 87.5%. ^{1}H NMR (600 MHz, $CDCl_3$) δ 8.33 (s, NH), 7.17–7.14 (m, 4H), 7.01 (d, *J* = 2.76, 2H), 6.85–6.82 (m, 4H), 3.50 (s, 6H). ^{13}C NMR (150 MHz, $CDCl_3$) δ 156.59 (2C), 130.78 (2CH), 126.97 (2CH), 126.07 (2C), 120.31 (2CH), 120.22 (2C), 118.06 (2CH), 110.81 (2CH), 55.13 (CH_3), 55.10 (CH_3). HRMS (ESI) m/z: calcd for M^+ $C_{18}H_{18}O_2N$, 280.1332; found, M^+ 280.1334.

1.2.2. 3,4-bis(3-methoxyphenyl)-1*H*-pyrrole (**5b**)

Sticky liquid 0.51 g. Yield 89.5%. ^{1}H NMR (600MHz, $CDCl_3$) δ 8.48 (s, NH), 7.21–7.18 (t, *J* = 7.9 Hz, 2H), 6.92–6.90 (m, 4H), 6.86 (s, 2H), 6.77–6.75 (dd, *J* = 8.2, 2.0Hz, 2H), 3.70 (s, 6H). ^{13}C NMR (150 MHz, $CDCl_3$) δ 159.26 (2C), 137.06 (2C), 129.03 (2CH), 123.36 (2C), 121.10 (2CH), 117.43 (2CH), 113.79 (2CH), 111.58 (2CH), 55.01 (2CH_3). HRMS (ESI) m/z: calcd for M^+ $C_{18}H_{18}O_2N$, 280.1332; found, M^+ 280.1335.

1.2.3. 3,4-bis(4-methoxyphenyl)-1*H*-pyrrole (**5c**)

White solid 0.52 g. Yield 91.2%. ^{1}H NMR (600 MHz, $CDCl_3$) δ 8.26 (s, NH), 7.22–7.20 (d, *J*=8.6Hz, 4H), 6.85–6.82 (m, 6H), 3.81 (s, 6H). ^{13}C NMR (150 MHz, $CDCl_3$) δ 157.74 (2C), 129.55 (4CH), 128.33 (2C), 123.00 (2C), 116.72 (2CH), 113.54 (4CH), 55.17 (2CH_3). HRMS (ESI) m/z: calcd for M^+ $C_{18}H_{18}O_2N$, 280.1332; found, M^+ 280.1338.

1.2.4. 3,4-bis(3,4-dimethoxyphenyl)-1*H*-pyrrole (**5d**)

Yellowish solid 0.63 g. Yield 92.6%. ^{1}H NMR (500 MHz, $CDCl_3$) δ (ppm) 8.41 (s, NH), 6.89-6.88 (d, *J* = 2.6 Hz, 2H), 6.87-6.85 (dd, *J* = 8.2, 1.8 Hz, 2H), 6.81-6.80 (m, 4H), 3.87 (s, 6H), 3.69 (s, 6H). ^{13}C NMR (125 MHz, $CDCl_3$) δ (ppm) 148.29 (2C), 147.10 (2C), 128.57 (2C), 123.11 (2C), 120.55 (2CH), 116.72 (2CH), 112.18 (2CH), 110.99 (2CH), 55.81 (2CH_3), 55.57 (2CH_3). HRMS (ESI) m/z: calcd for M^+ $C_{20}H_{22}O_4N$, 340.1543; found, M^+ 340.1543.

1.2.5. 3,4-bis(3,4,5-trimethoxyphenyl)-1*H*-pyrrole (**5e**)

Yellowish solid 0.73 g. Yield 91.2%. ^{1}H NMR (500 MHz, $CDCl_3$) δ (ppm) 8.52 (s, NH), 6.94-6.93 (d, *J* = 2.6 Hz, 2H), 6.51 (s, 4H), 3.83 (s, 6H), 3.71 (s, 12H). ^{13}C NMR (125 MHz, $CDCl_3$) δ (ppm) 152.75 (4C), 136.13 (2C), 131.25 (2C), 123.44 (2C), 116.95 (2CH), 105.78 (4CH), 60.90 (2CH_3), 55.89 (4CH_3). HRMS (ESI) m/z: calcd for M^+ $C_{22}H_{26}O_6N$, 400.1755; found, M^+ 400.1759.

1.3. General procedure for the synthesis of acyl chloride **6a - 6c**

To a solution of the substituted benzoic acid (2.4 mmol) in anhydrous CH_2Cl_2 (10 mL) was added catalytic amount of DMF and oxalyl chloride (00.6 mL, 13.6 mmol) at room temperature under argon atmosphere. After stirring for 2 h, the solvent was removed by vacuum evaporation, and the compounds **6a - 6c** were directly used without further purification.

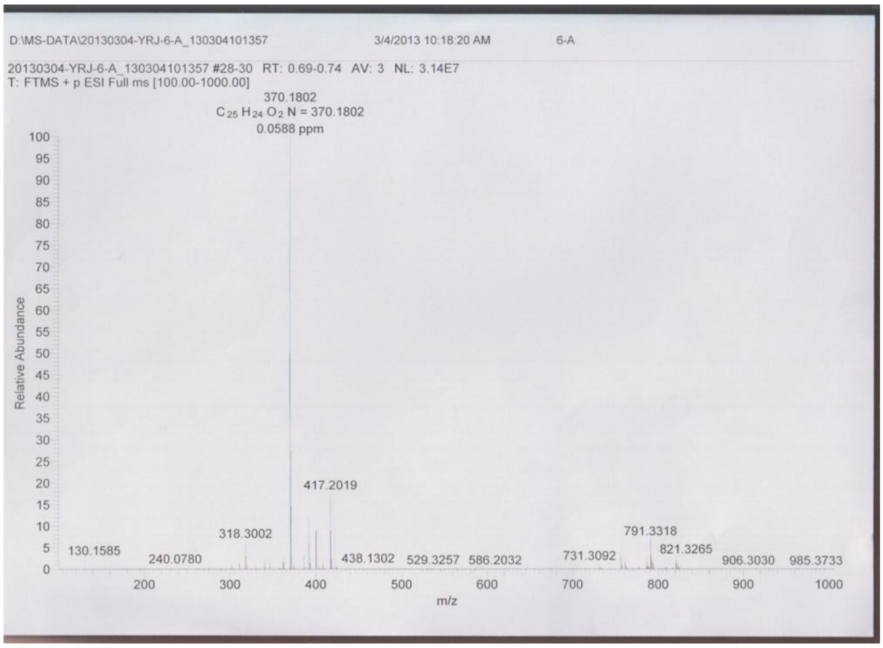

Figure S1. HRMS spectrum of **4a**

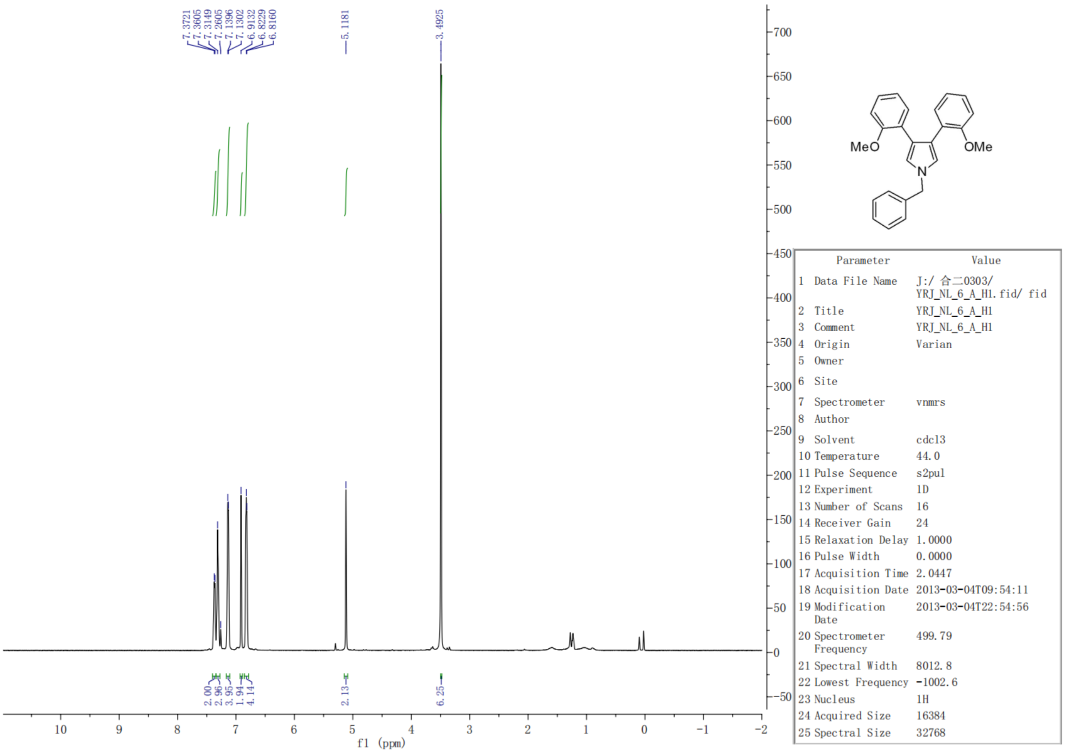

Figure S2. ^{1}H NMR spectrum of **4a**

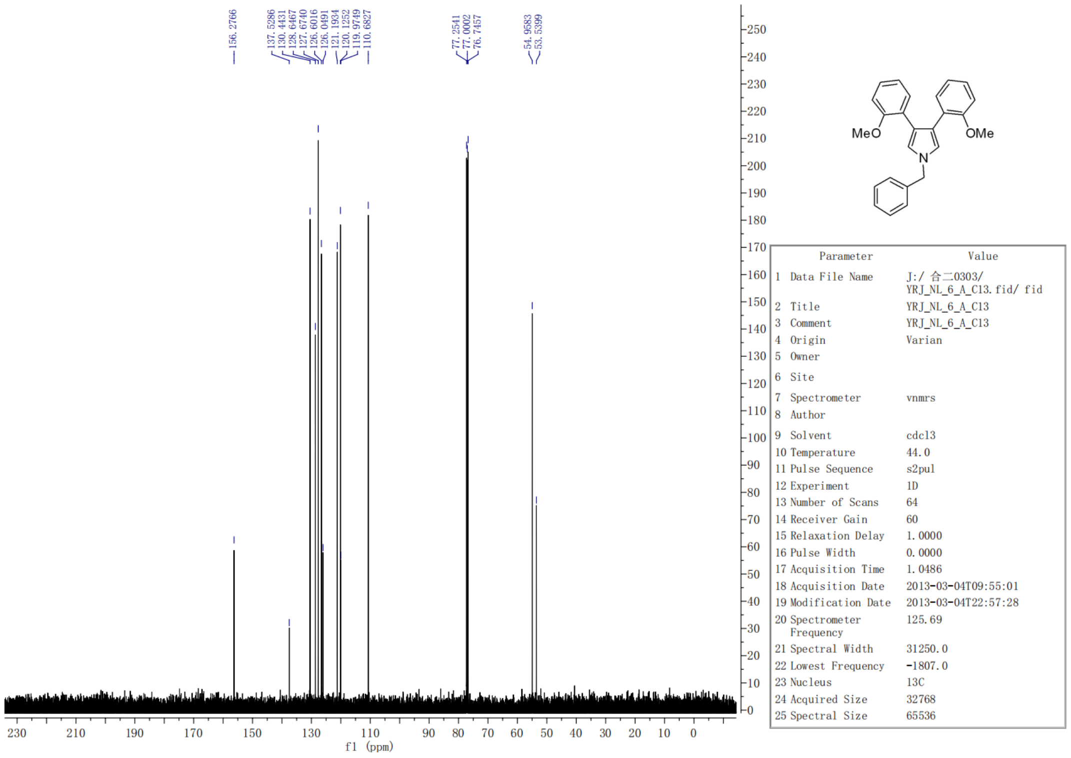

Figure S3. ^{13}C NMR spectrum of **4a**

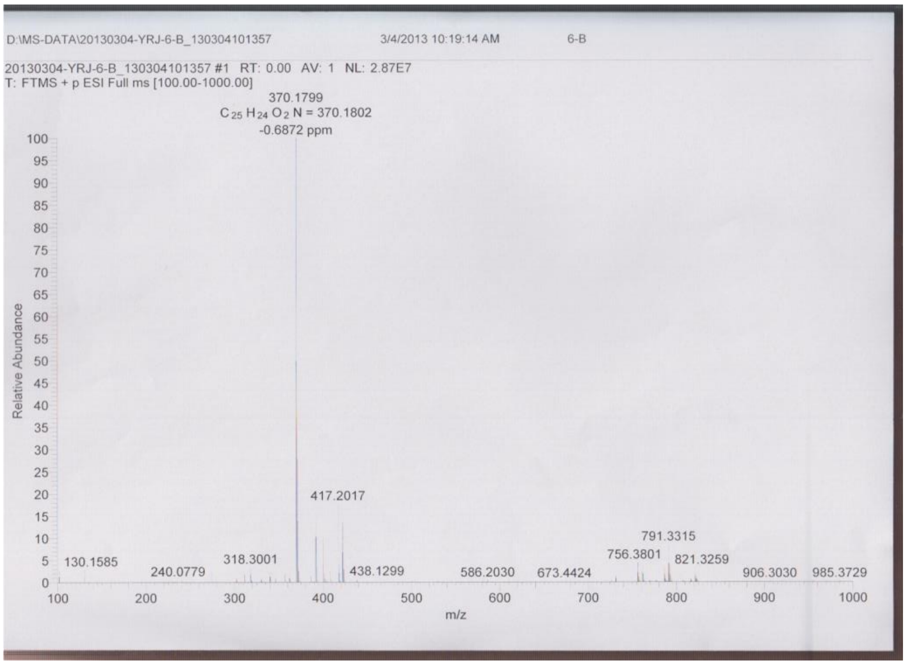

Figure S4. HRMS spectrum of **4b**

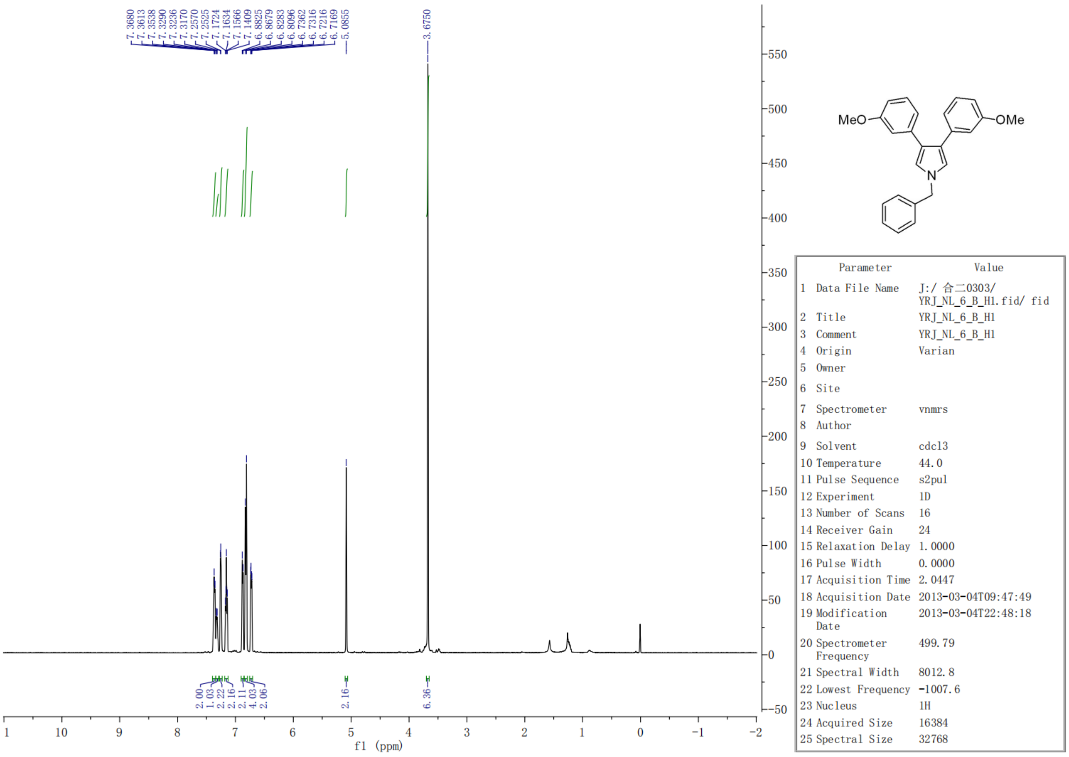

Figure S5. ^{1}H NMR spectrum of **4b**

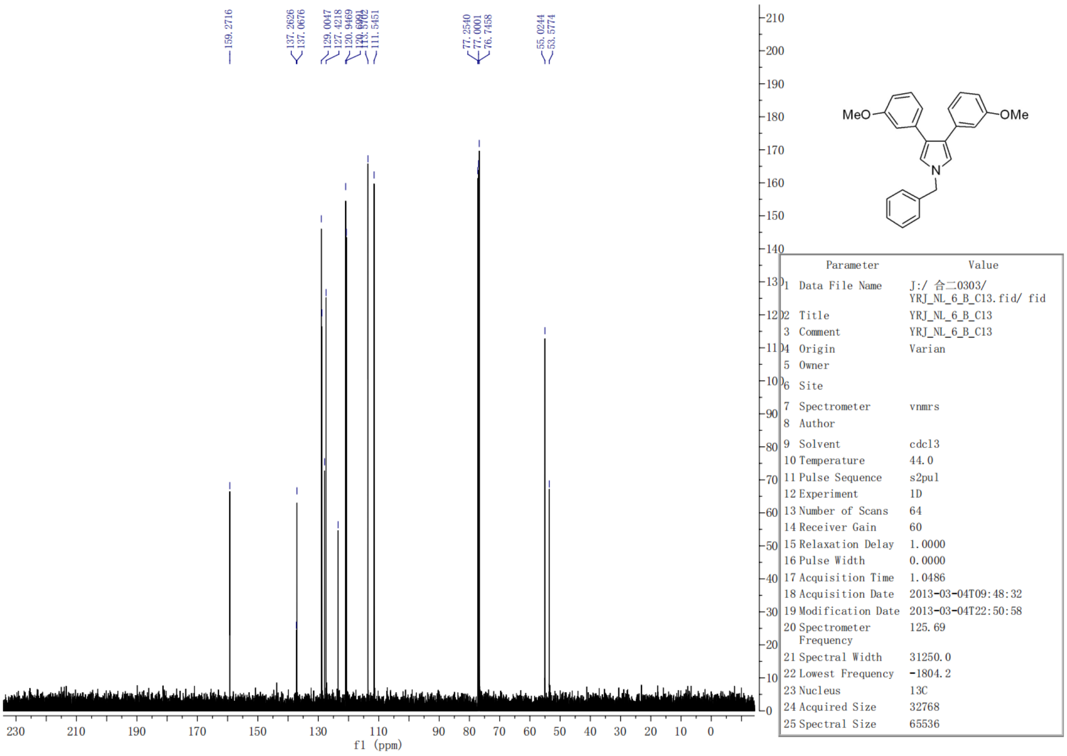

Figure S6. ^{13}C NMR spectrum of **4b**

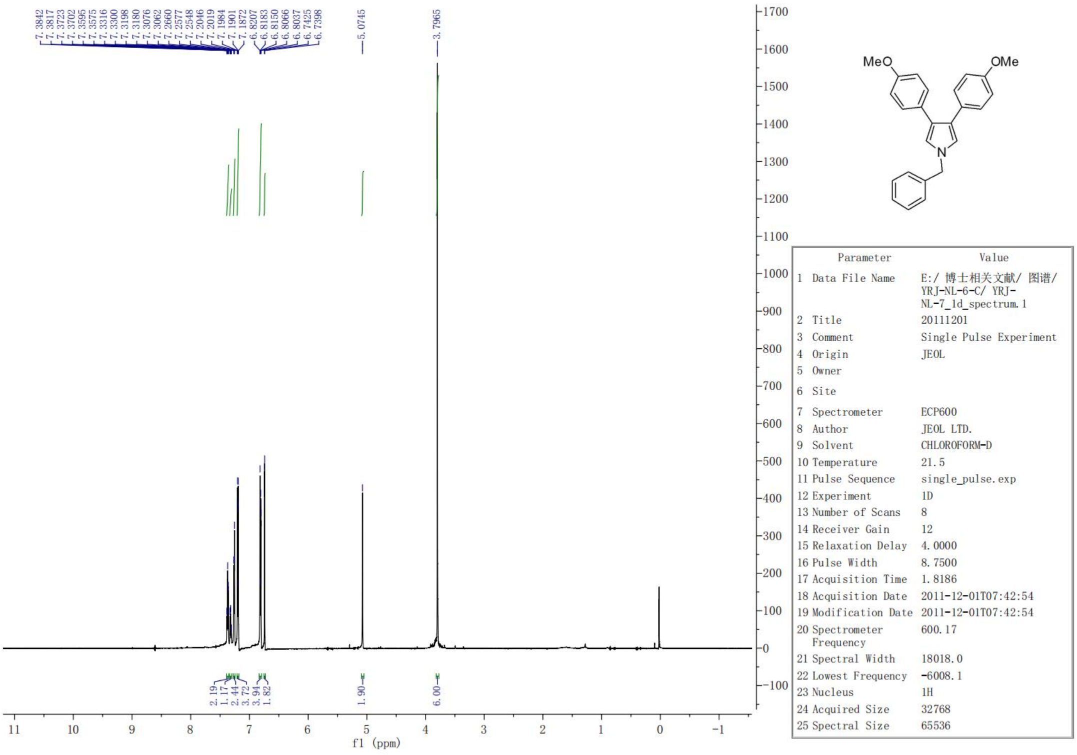

Figure S7. ^{1}H NMR spectrum of **4c**

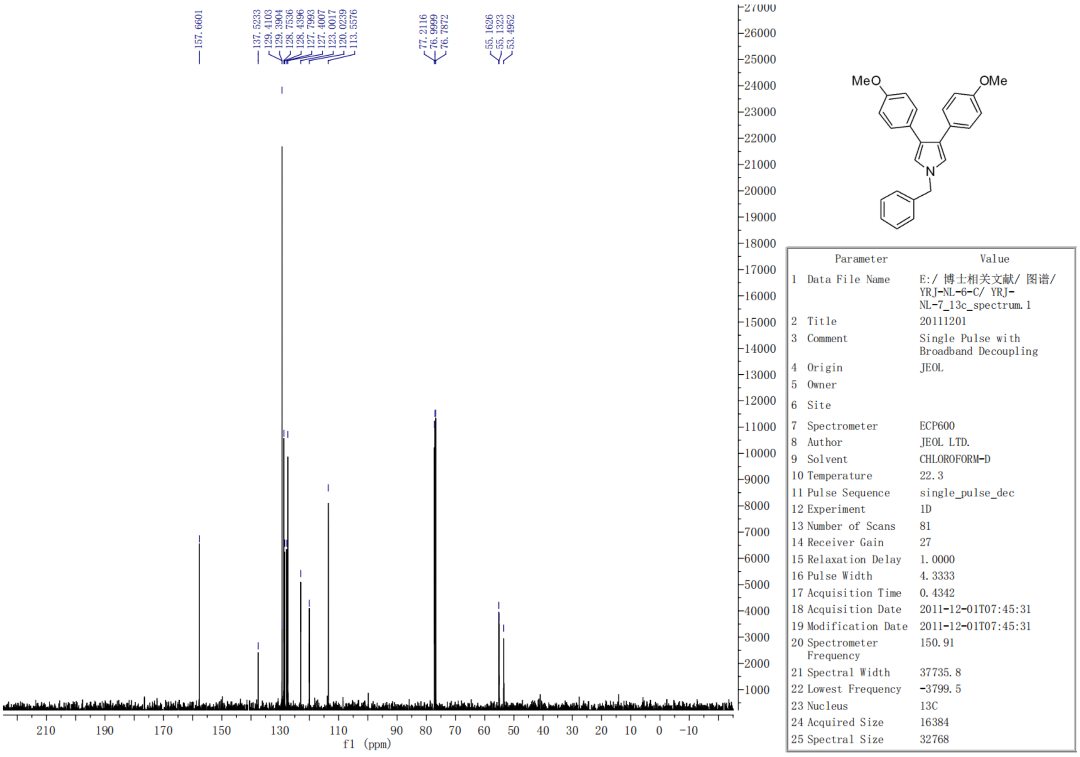

Figure S8. ^{13}C NMR spectrum of **4c**

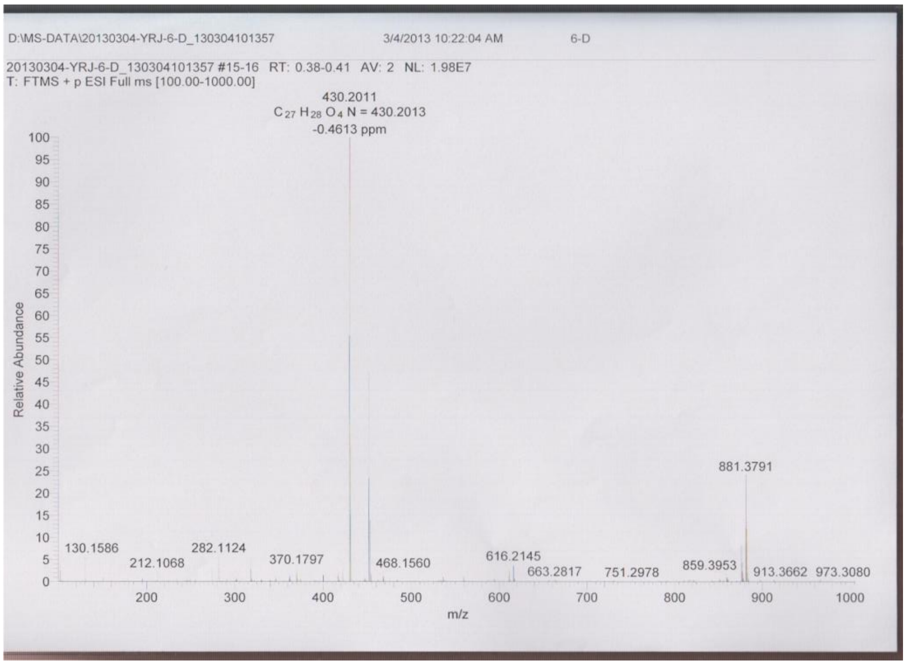

Figure S9. HRMS spectrum of **4d**

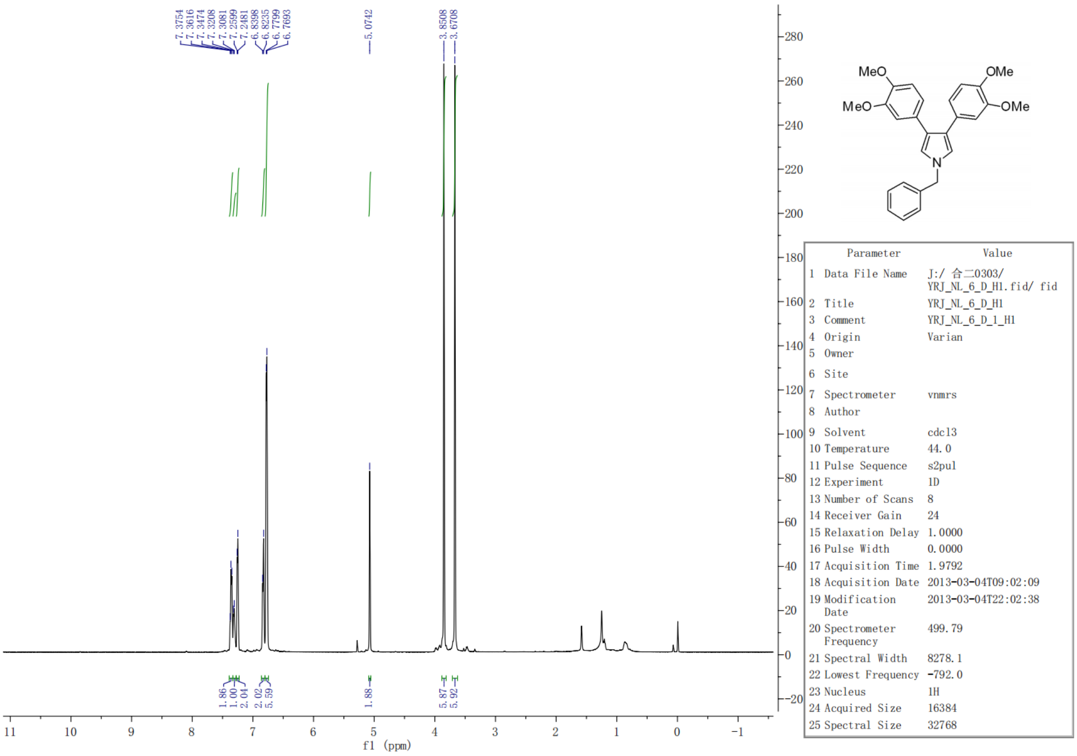

Figure S10. ^{1}H NMR spectrum of **4d**

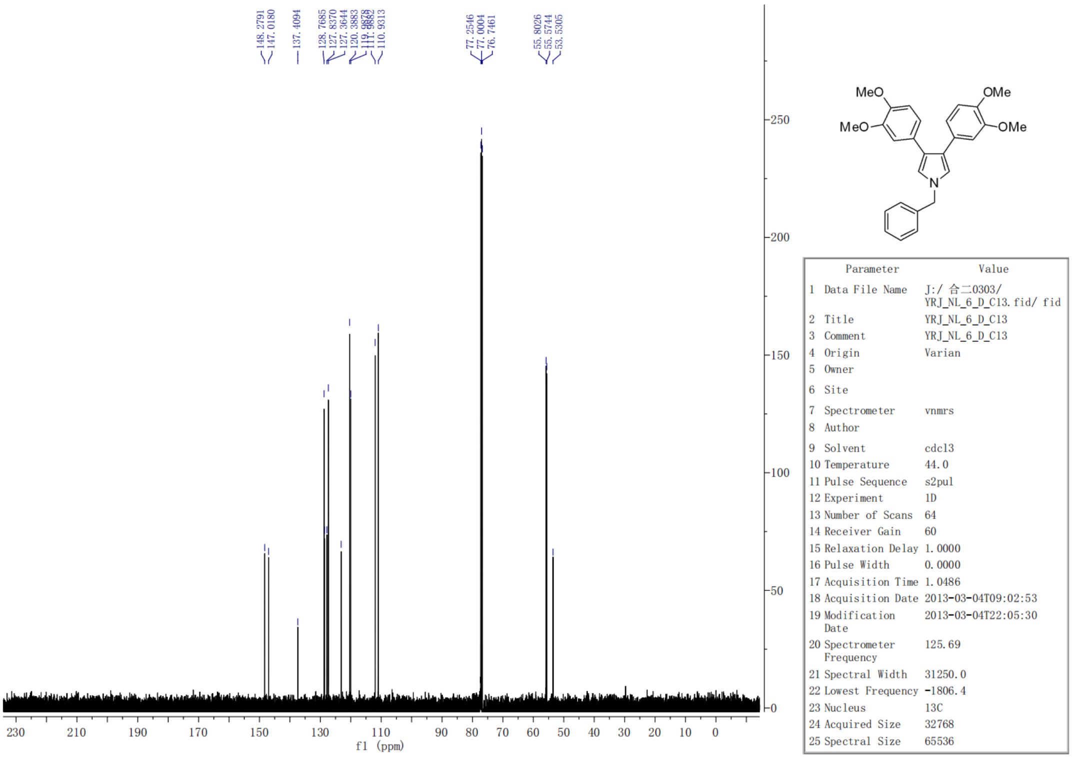

Figure S11. ^{13}C NMR spectrum of **4d**

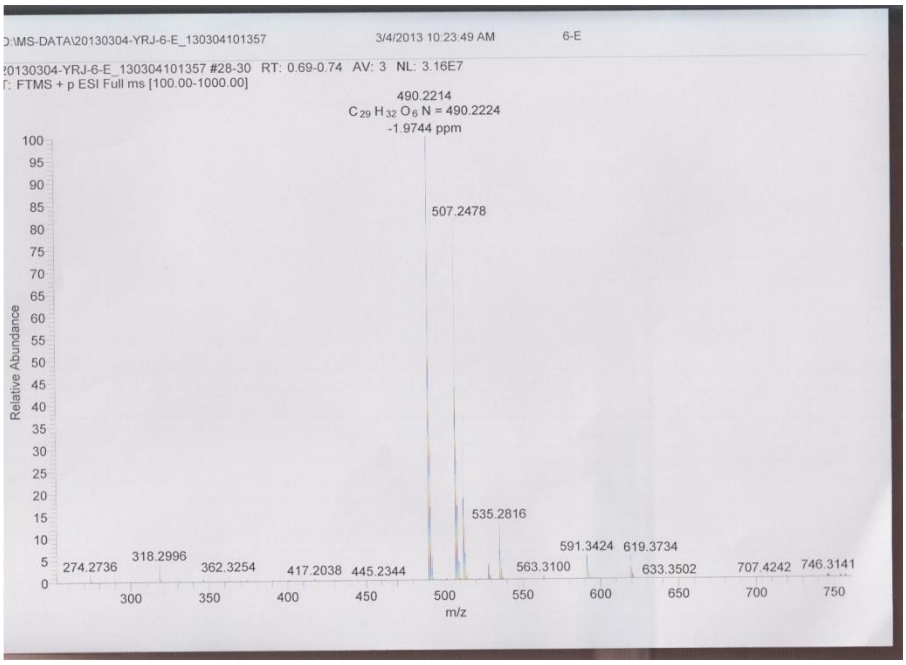

Figure S12. HRMS spectrum of **4e**

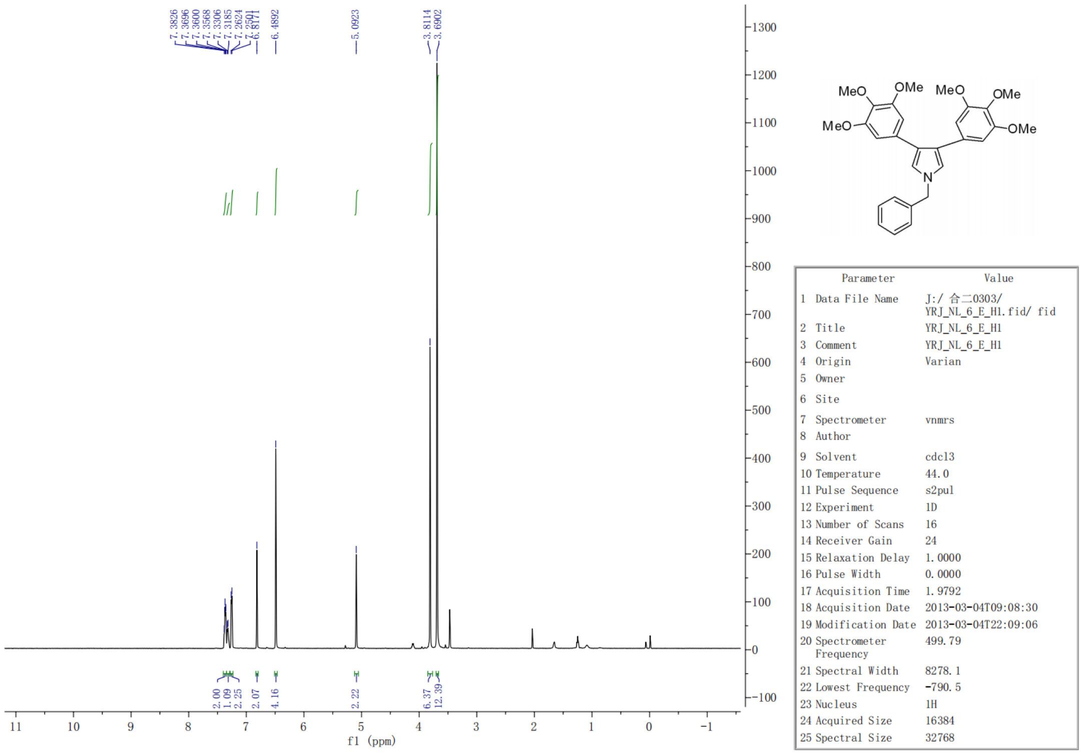

Figure S13. ^{1}H NMR spectrum of **4e**

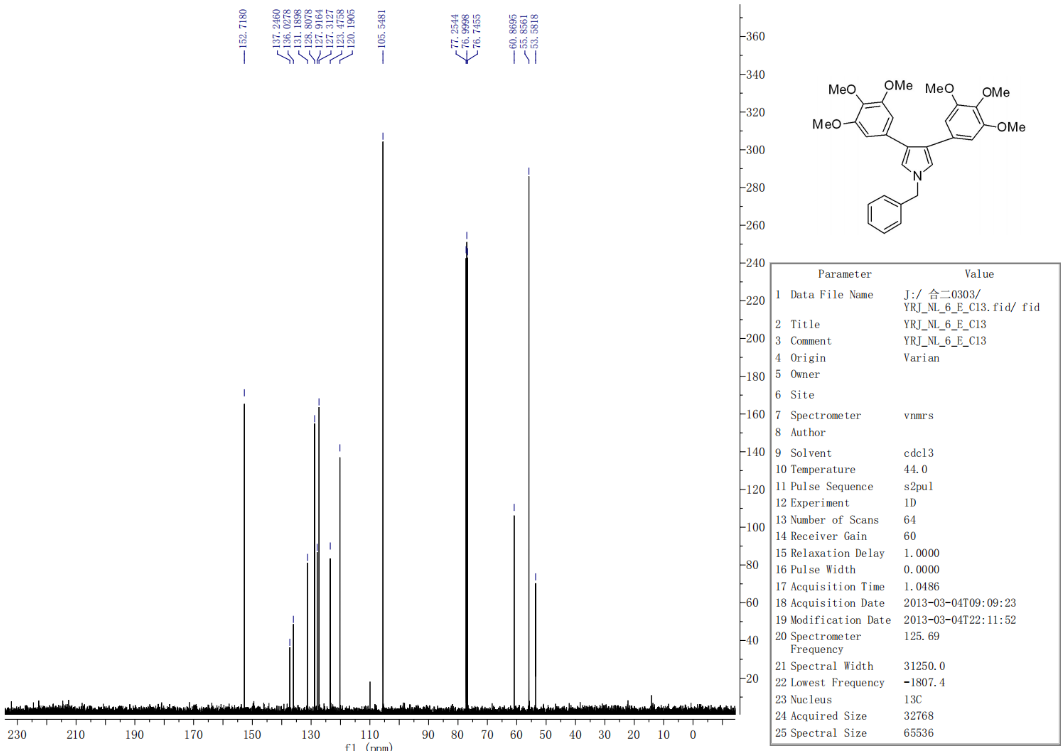

Figure S14. ^{13}C NMR spectrum of **4e**

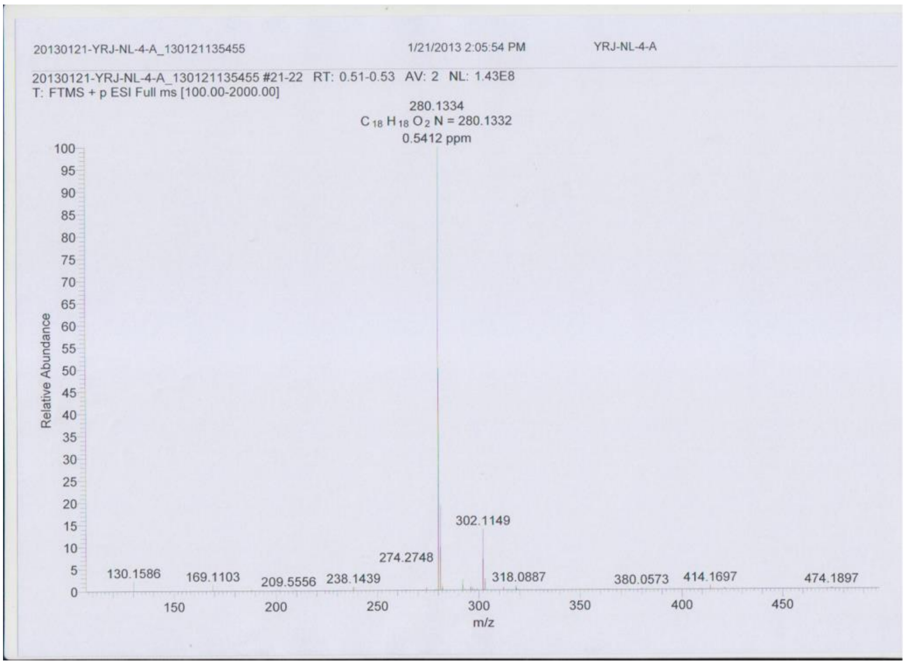

Figure S15. HRMS spectrum of **5a**

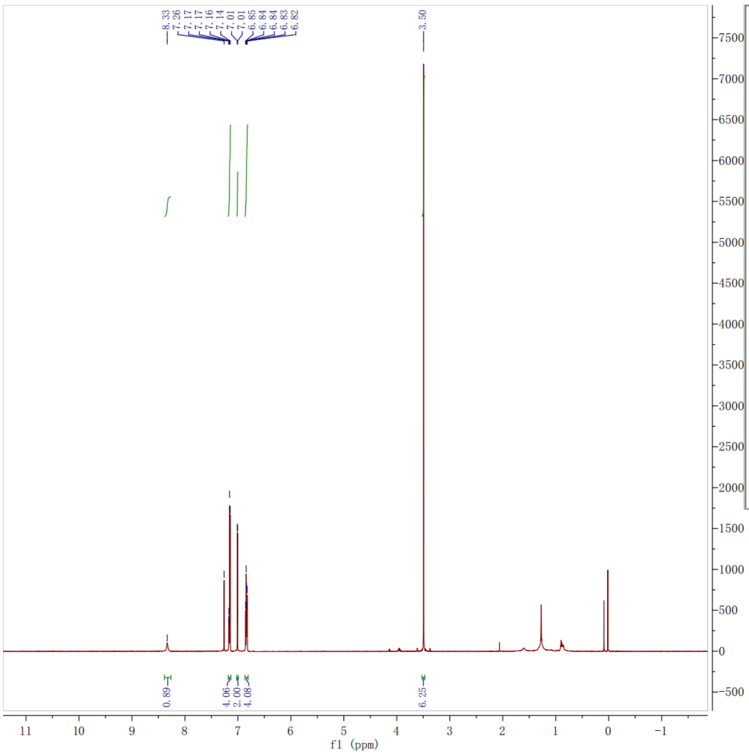

Parameter	Value
1 Data File Name	C:/ Users/ 洛璃/ OneDrive/ 桌面/ 原始谱图/ YRJ-NL-4-A/ YRJ-NL-5-D-1_1d_spectrum-4. jdf
2 Title	20111201
3 Comment	Single Pulse Experiment
4 Origin	JEOL
5 Owner	
6 Site	
7 Spectrometer	ECP600
8 Author	JEOL LTD.
9 Solvent	CHLOROFORM-D
10 Temperature	21.5
11 Pulse Sequence	single_pulse.exp
12 Experiment	1D
13 Number of Scans	8
14 Receiver Gain	13
15 Relaxation Delay	4.0000
16 Pulse Width	8.7500
17 Acquisition Time	1.8186
18 Acquisition Date	2011-12-01T07:30:33
19 Modification Date	2011-12-01T12:14:29
20 Spectrometer Frequency	600.17
21 Spectral Width	18017.5
22 Lowest Frequency	-6006.7
23 Nucleus	1H
24 Acquired Size	32768
25 Spectral Size	32768

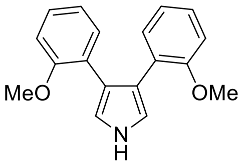

Figure S16. ^{1}H NMR spectrum of **5a**

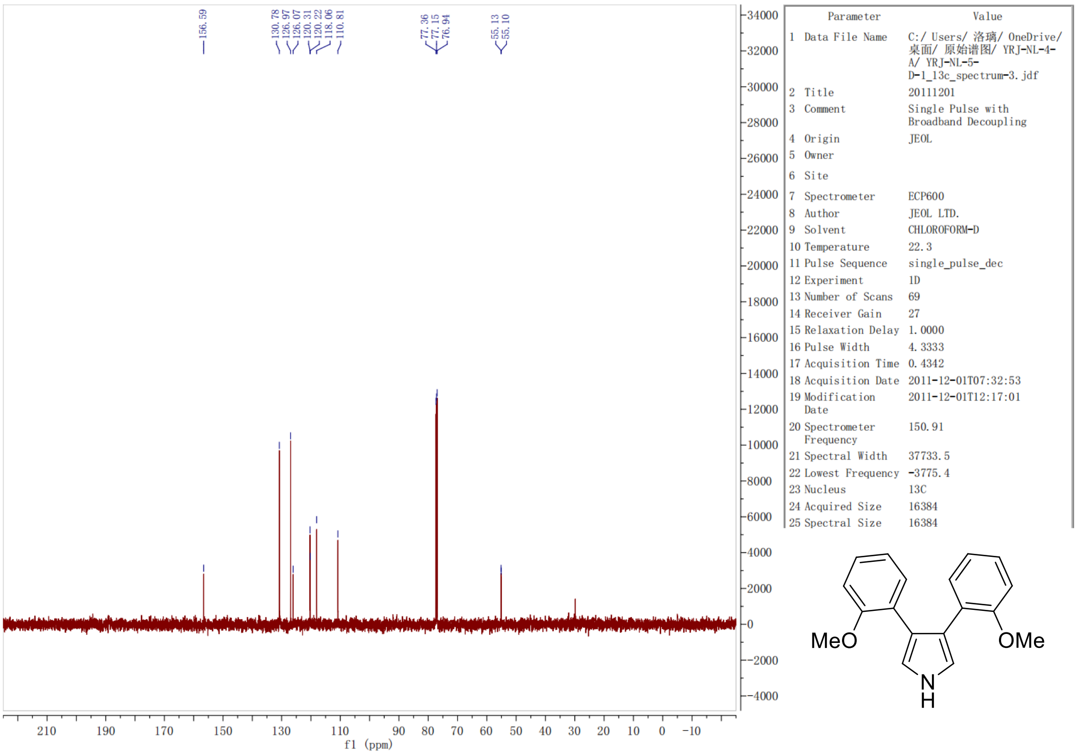

Figure S17. ^{13}C NMR spectrum of **5a**

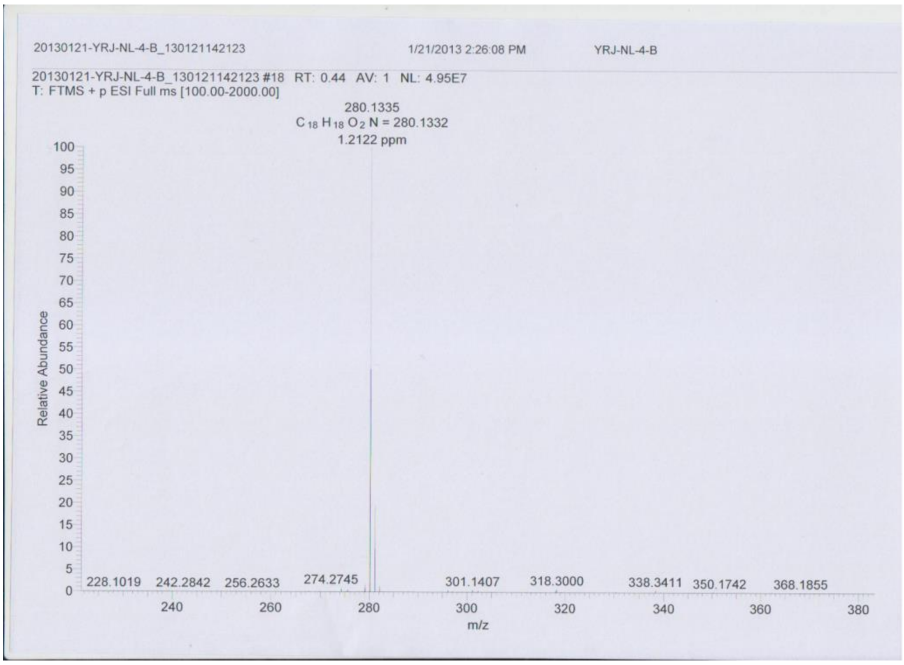

Figure S18. HRMS spectrum of **5b**

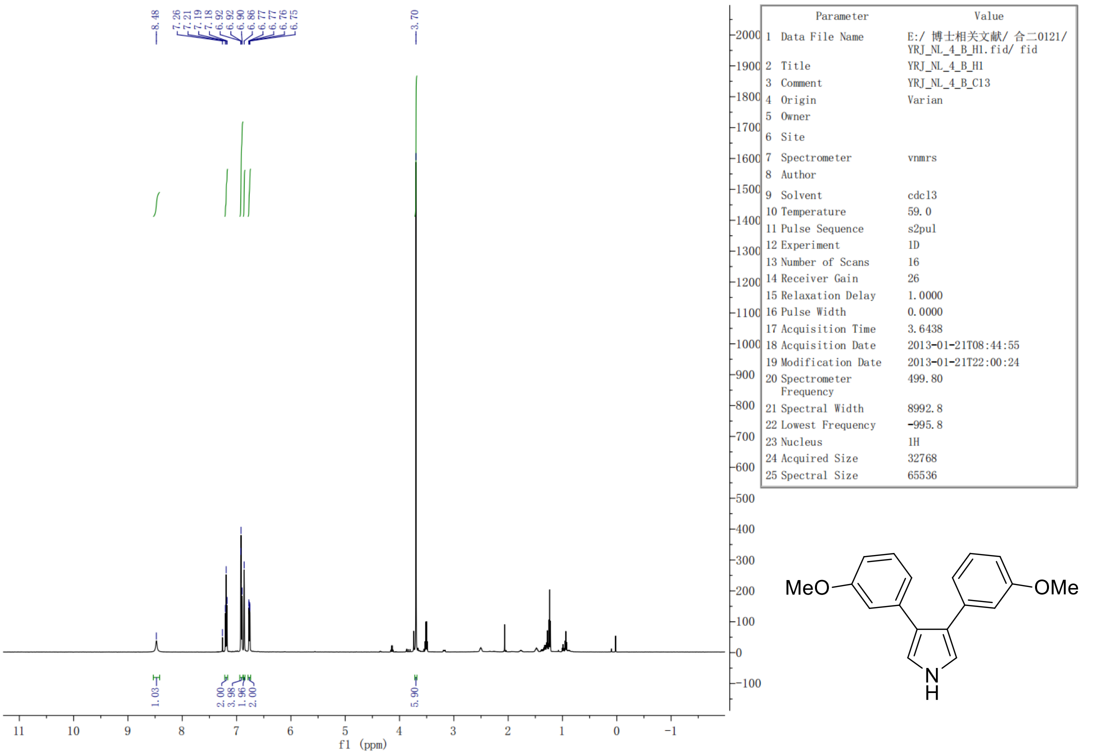

Figure S19. ^{1}H NMR spectrum of **5b**

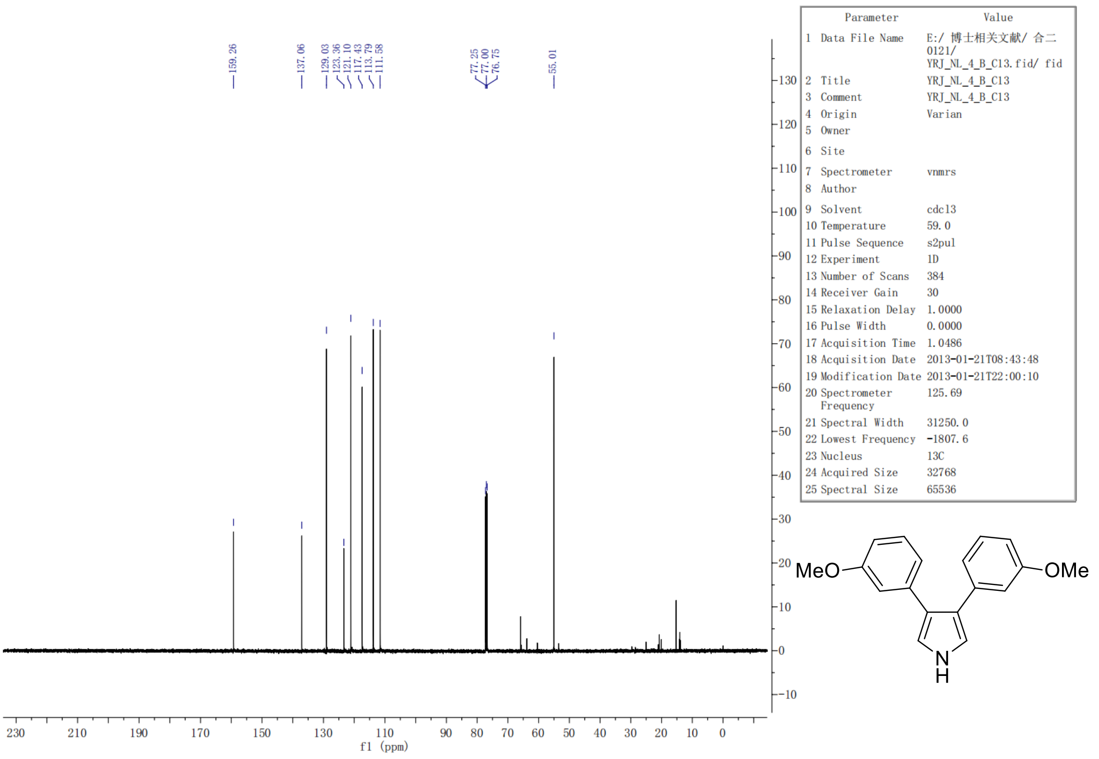

Figure S20. ^{13}C NMR spectrum of **5b**

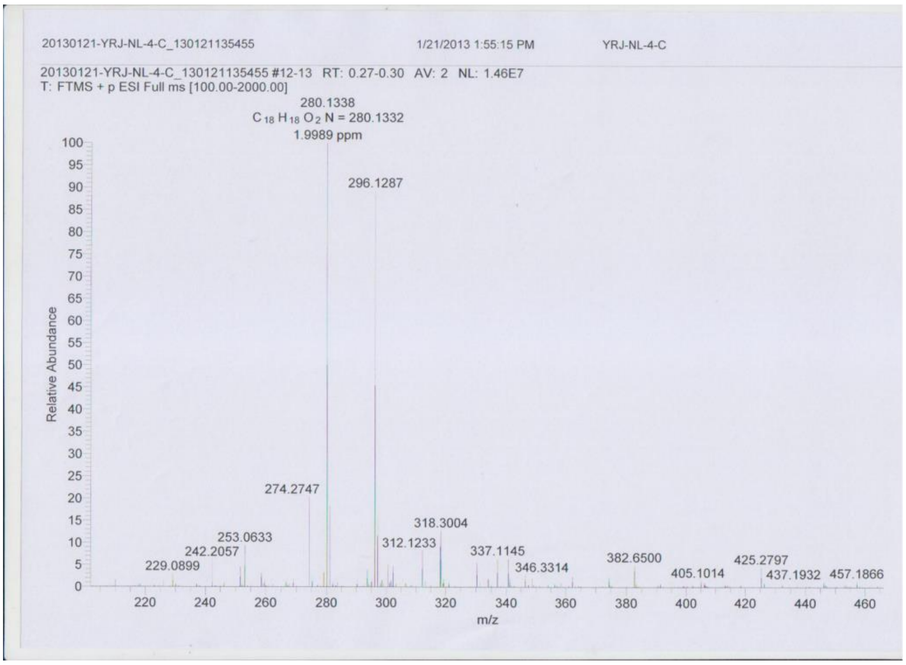

Figure S21. HRMS spectrum of **5c**

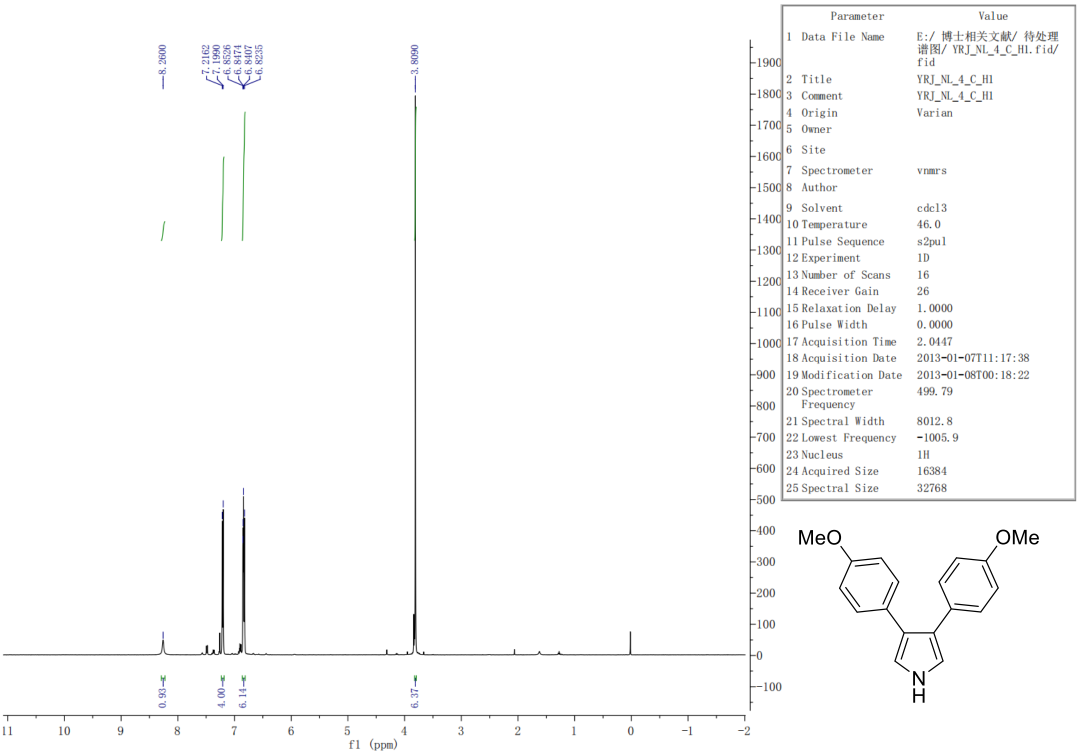

Figure S22. ^{1}H NMR spectrum of **5c**

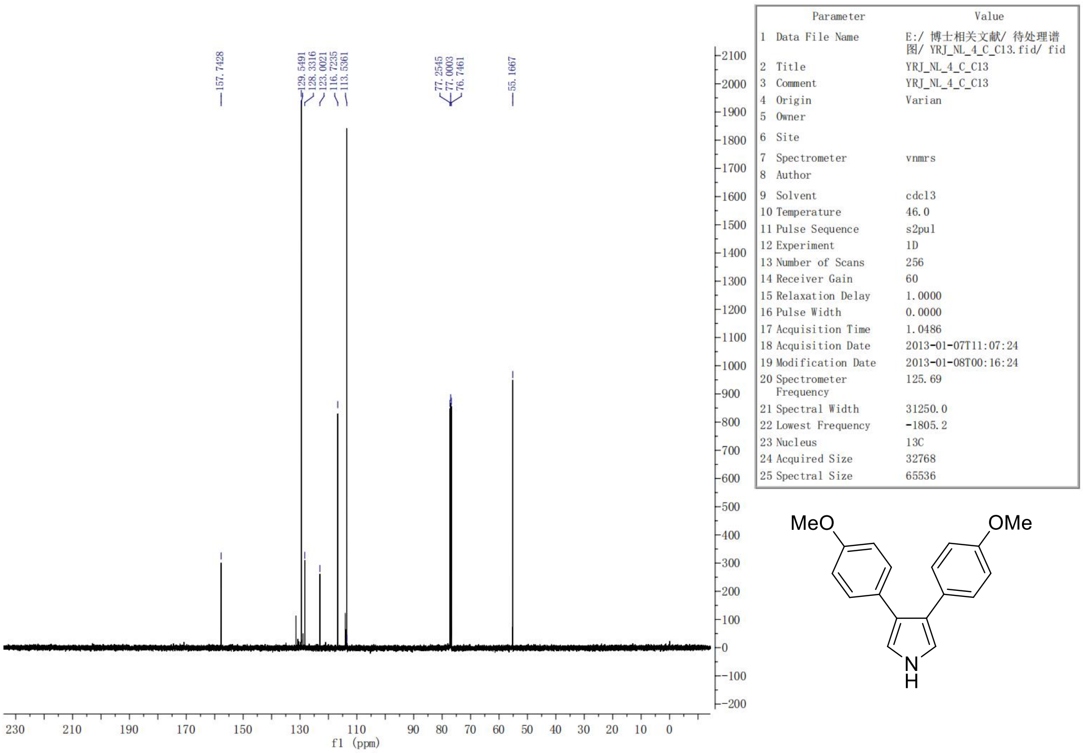

Figure S23. ^{13}C NMR spectrum of **5c**

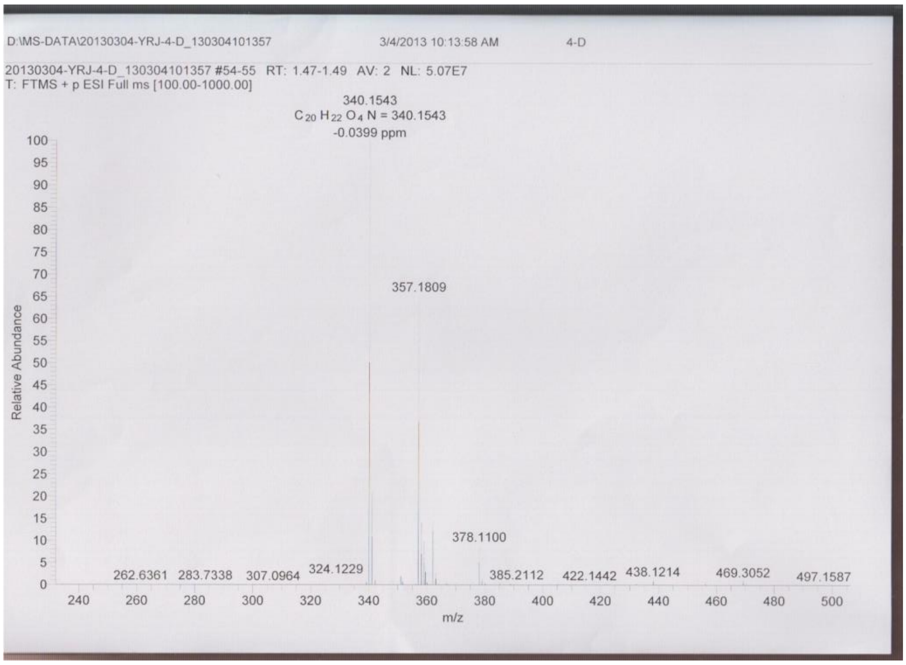

Figure S24. HRMS spectrum of **5d**

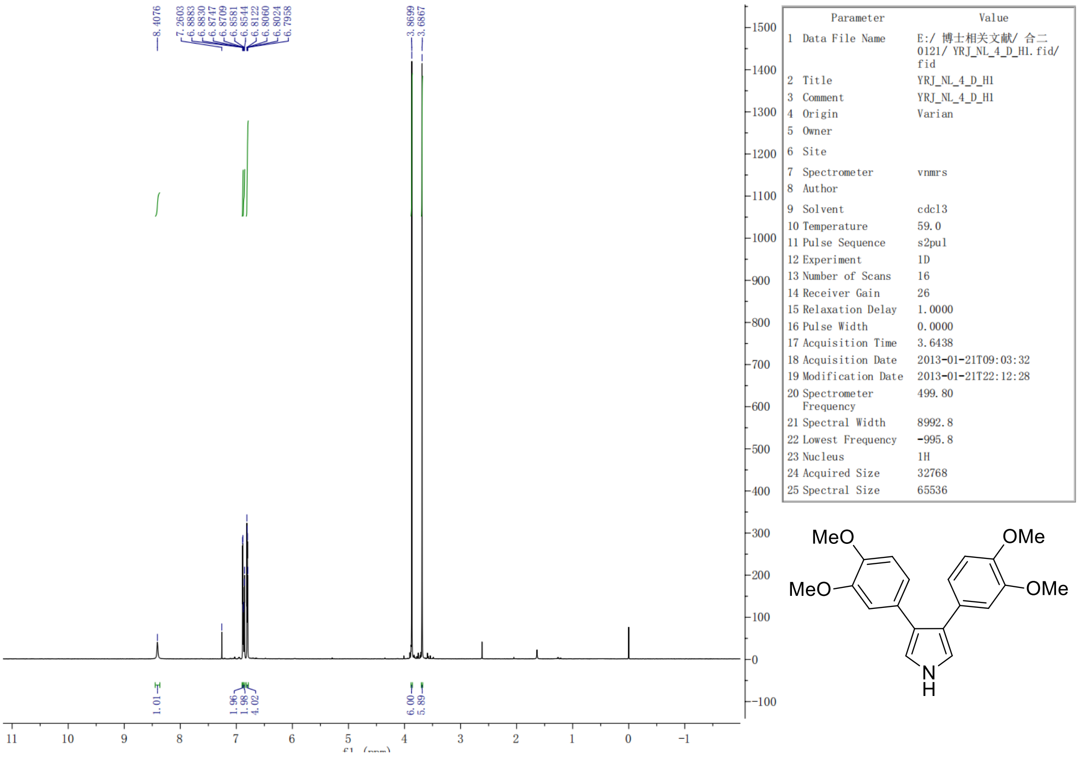

Figure S25. ^{1}H NMR spectrum of **5d**

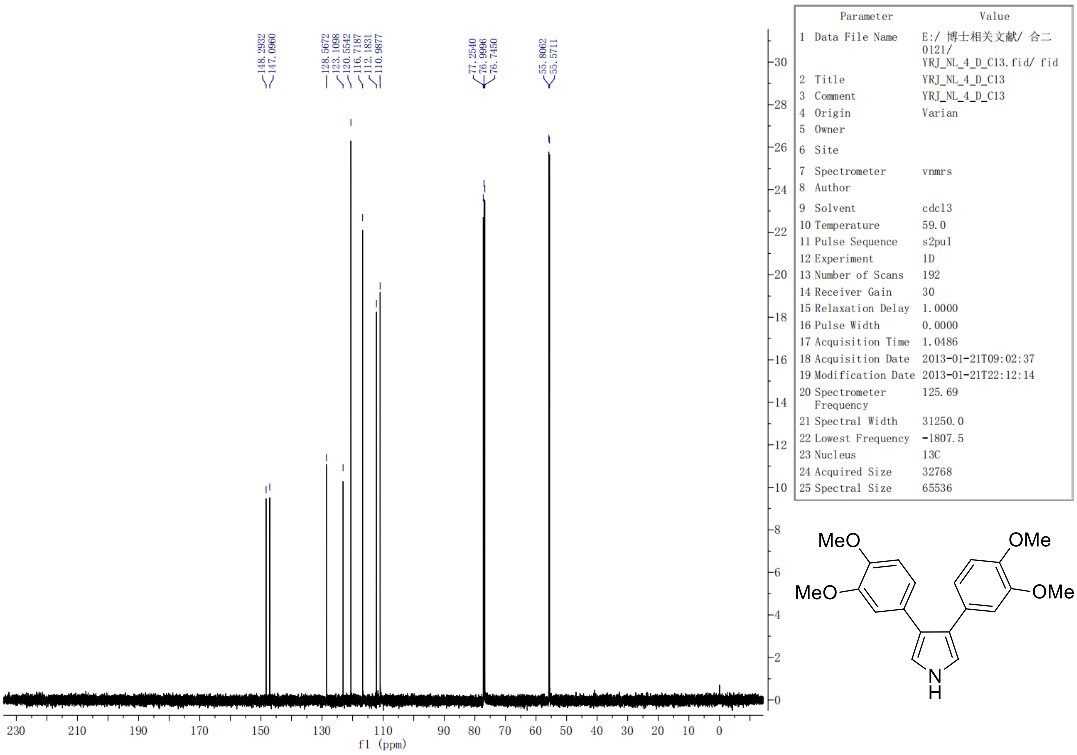

Figure S26. ^{13}C NMR spectrum of **5d**

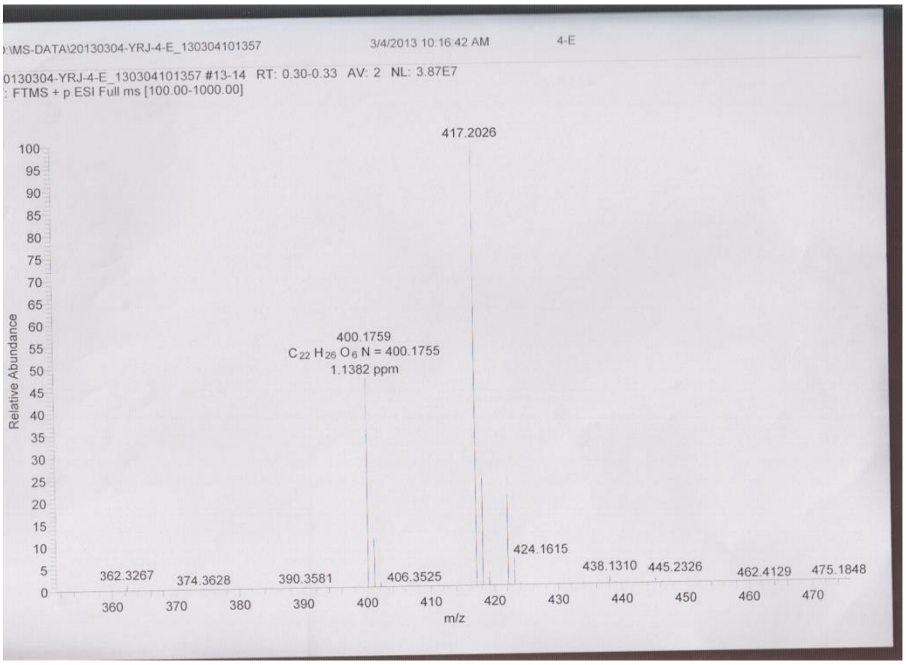

Figure 27. HRMS spectrum of **5e**

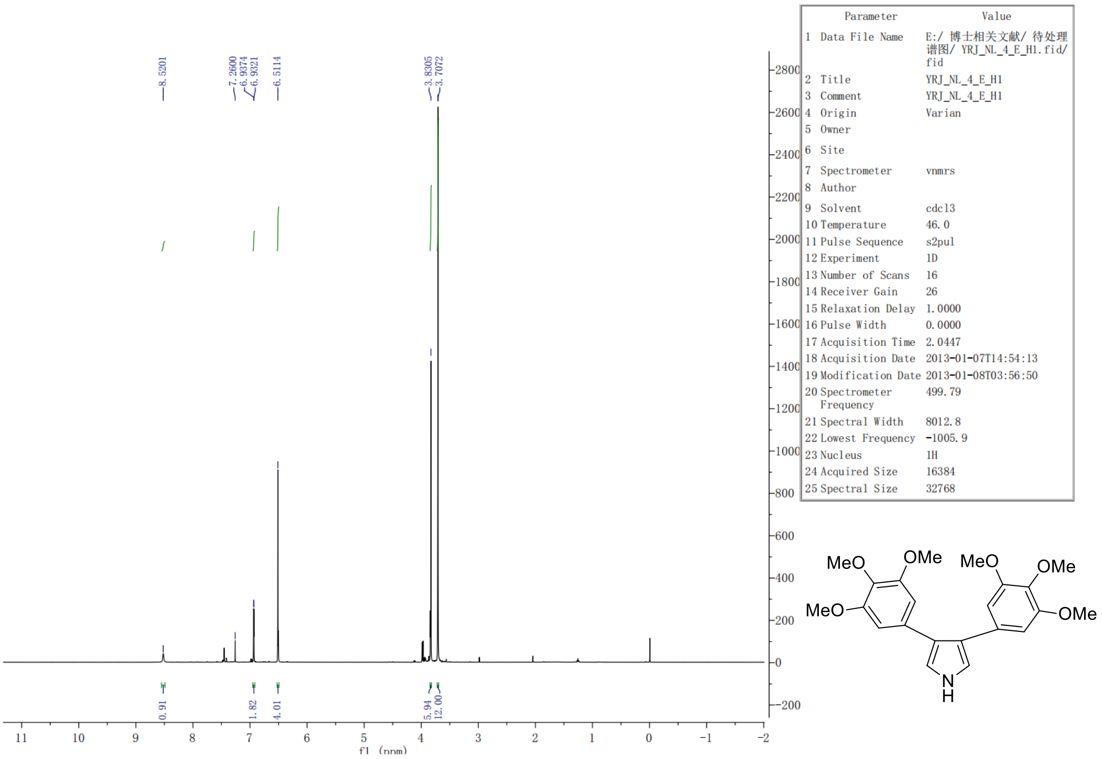

Figure S28. ^{1}H NMR spectrum of **5e**

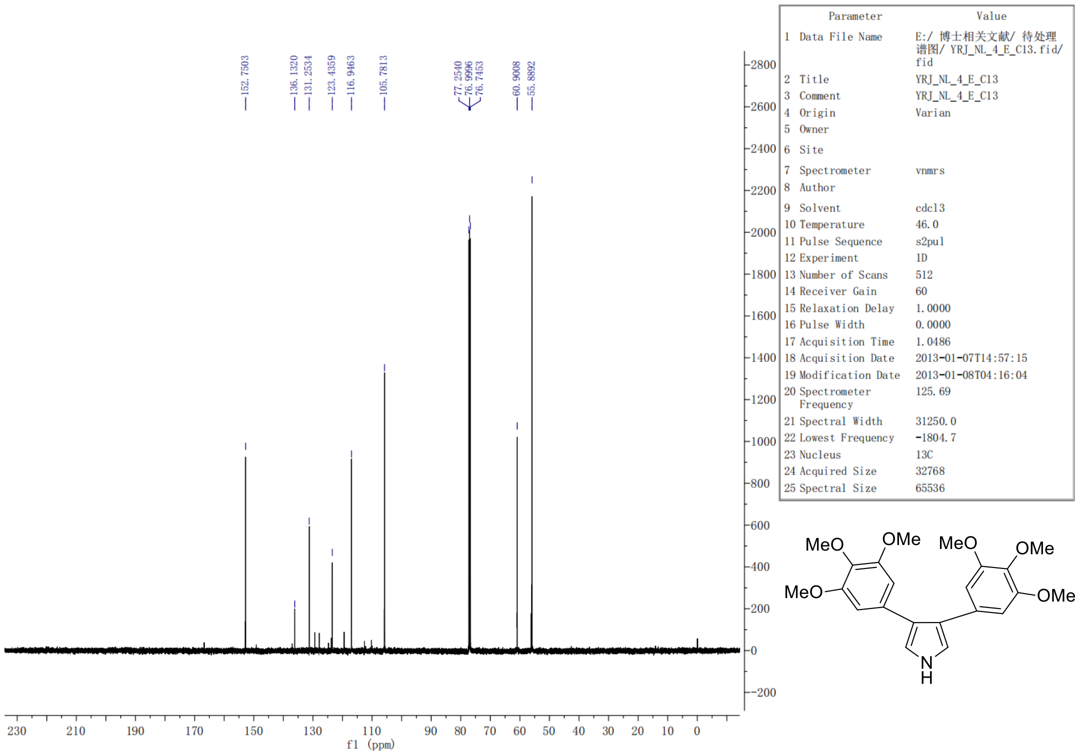

Figure S29. ^{13}C NMR spectrum of **5e**

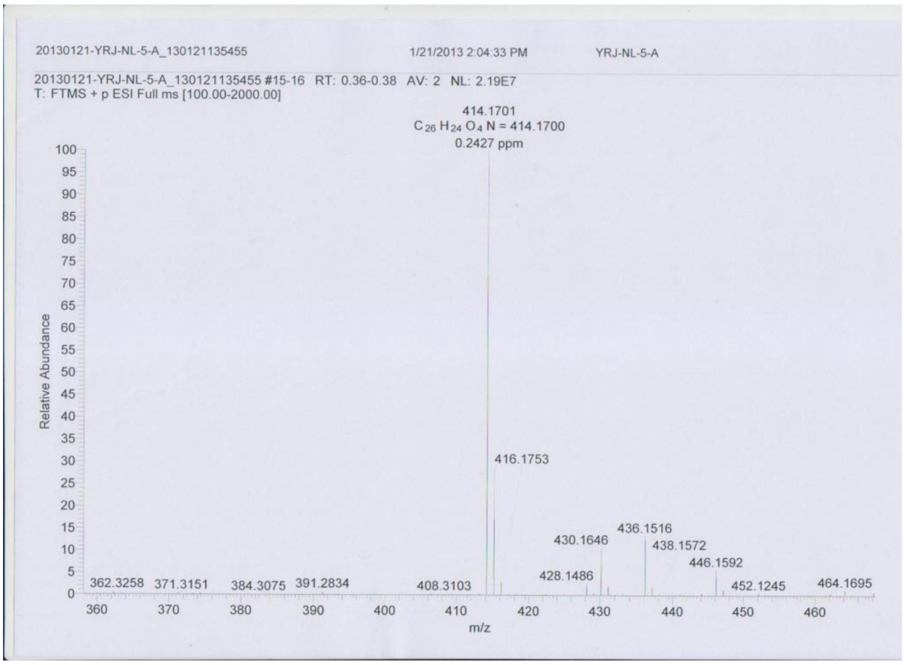

Figure S30. HRMS spectrum of **1a**

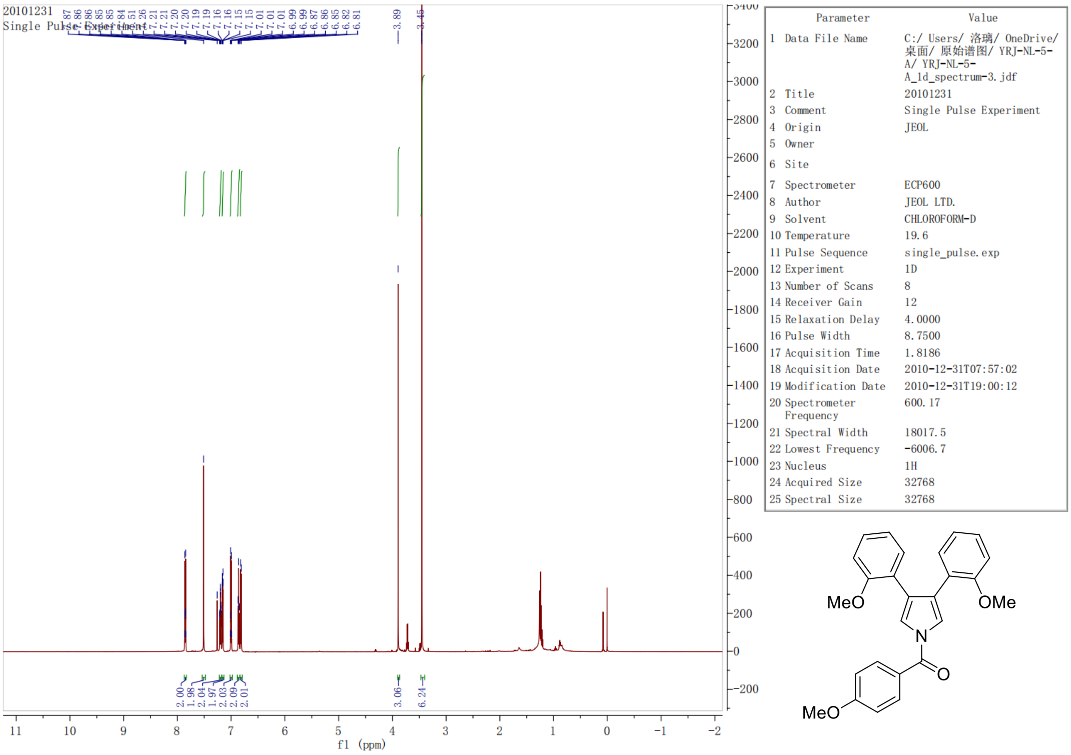

Figure S31. ^{1}H NMR spectrum of **1a**

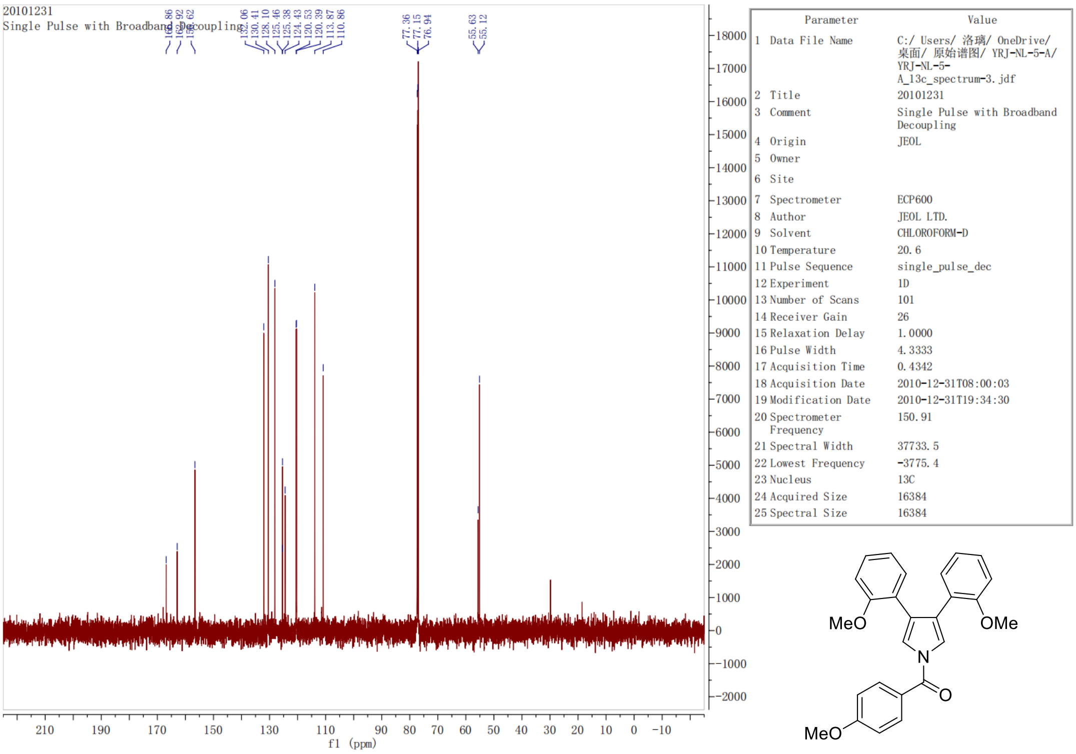

Figure S32. ^{13}C NMR spectrum of **1a**

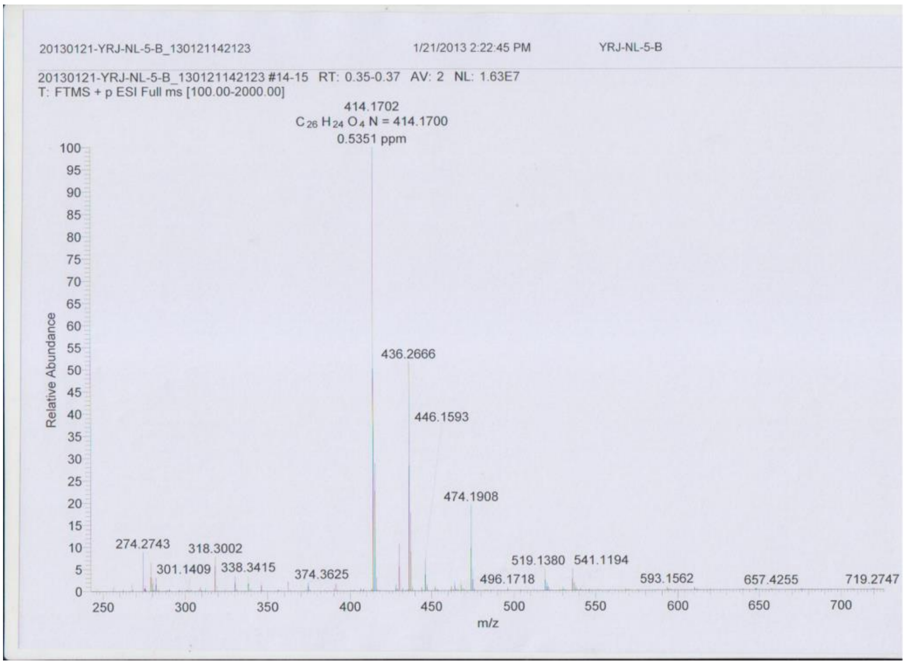

Figure S33. HRMS spectrum of **1b**

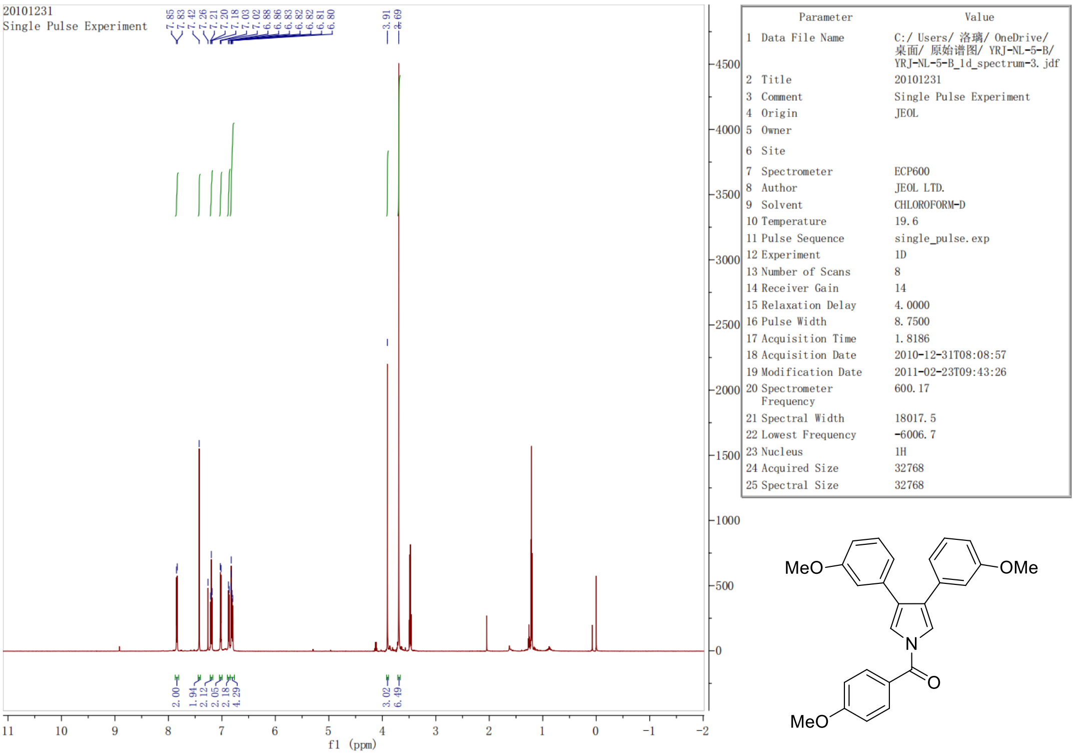

Figure S34. ^{1}H NMR spectrum of **1b**

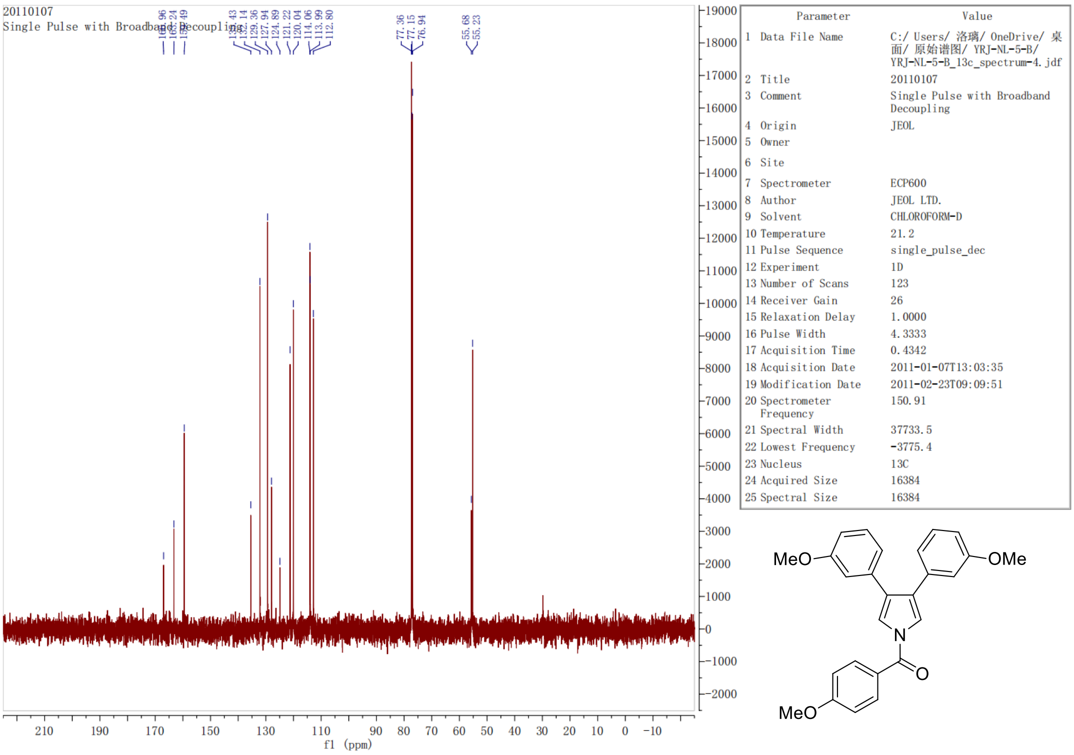

Figure S35. ^{13}C NMR spectrum of **1b**

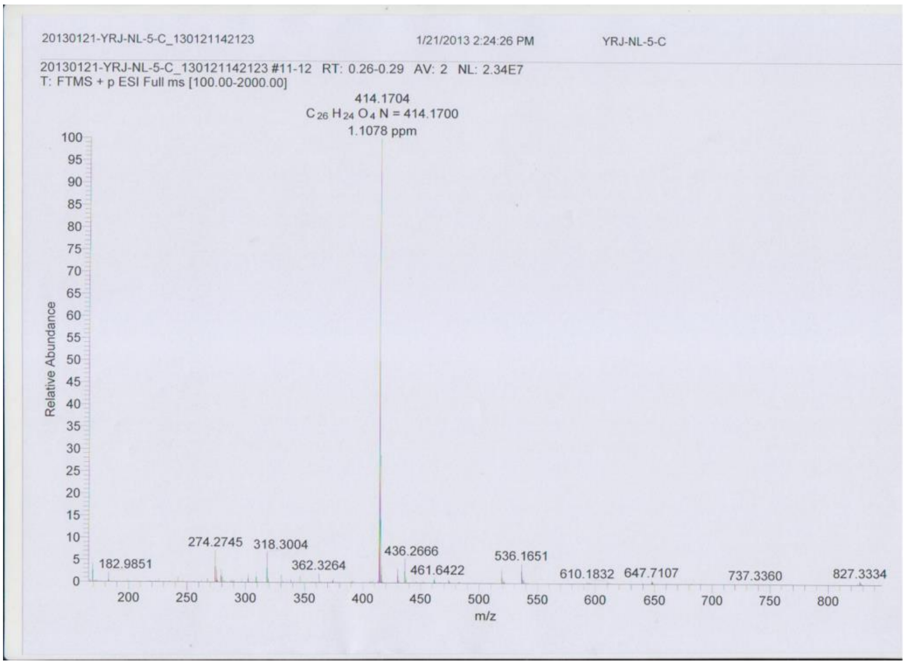

Figure S36. HRMS spectrum of **1c**

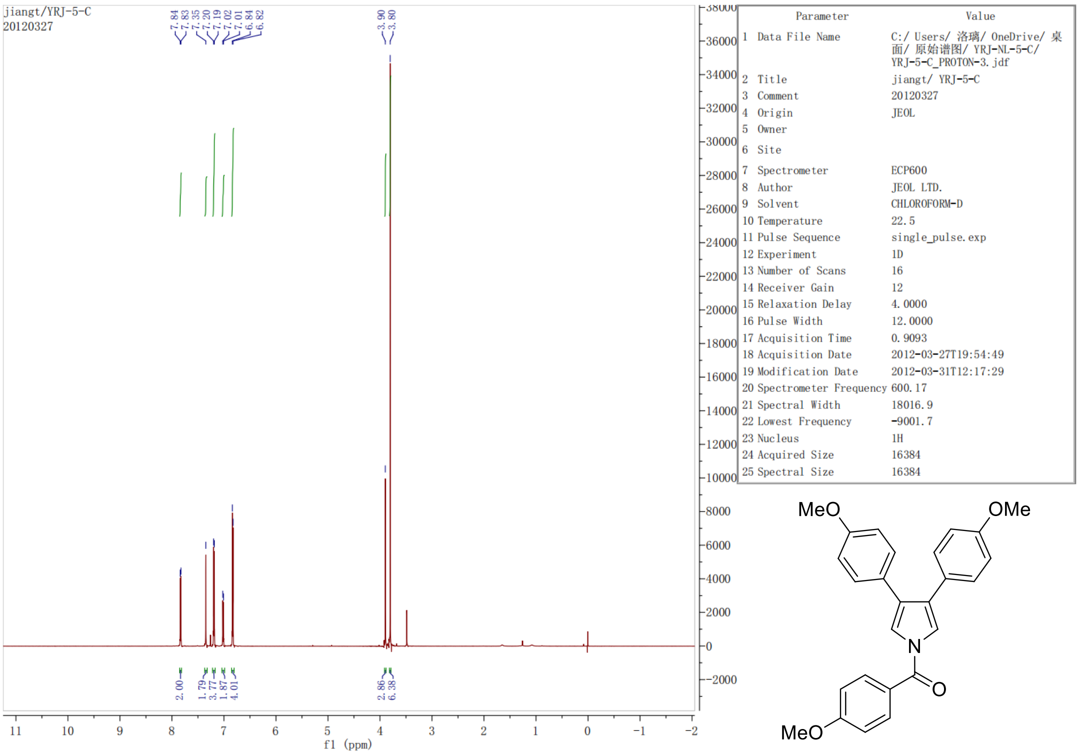

Figure S37. ^{1}H NMR spectrum of **1c**

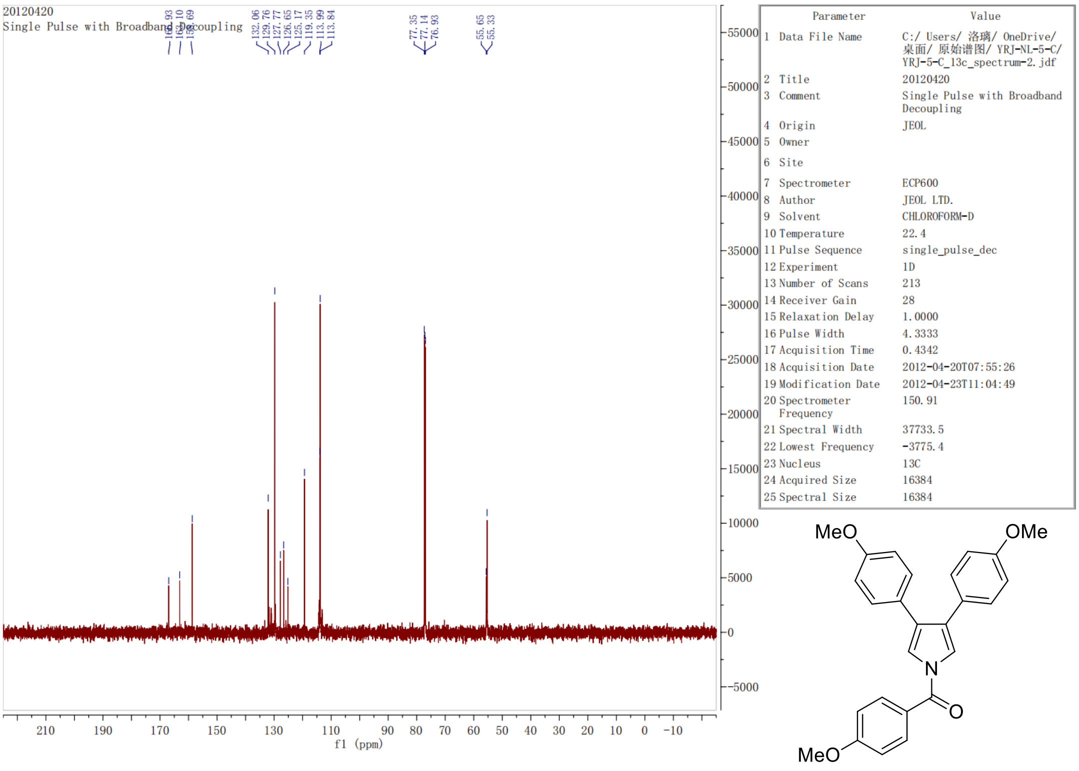

Figure S38. ^{13}C NMR spectrum of **1c**

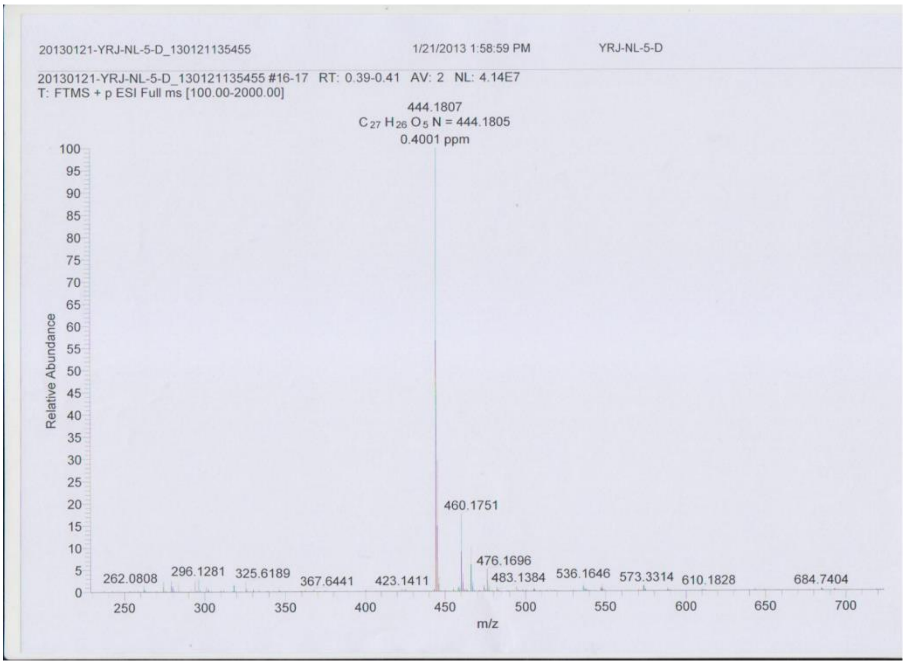

Figure S39. HRMS spectrum of **1d**

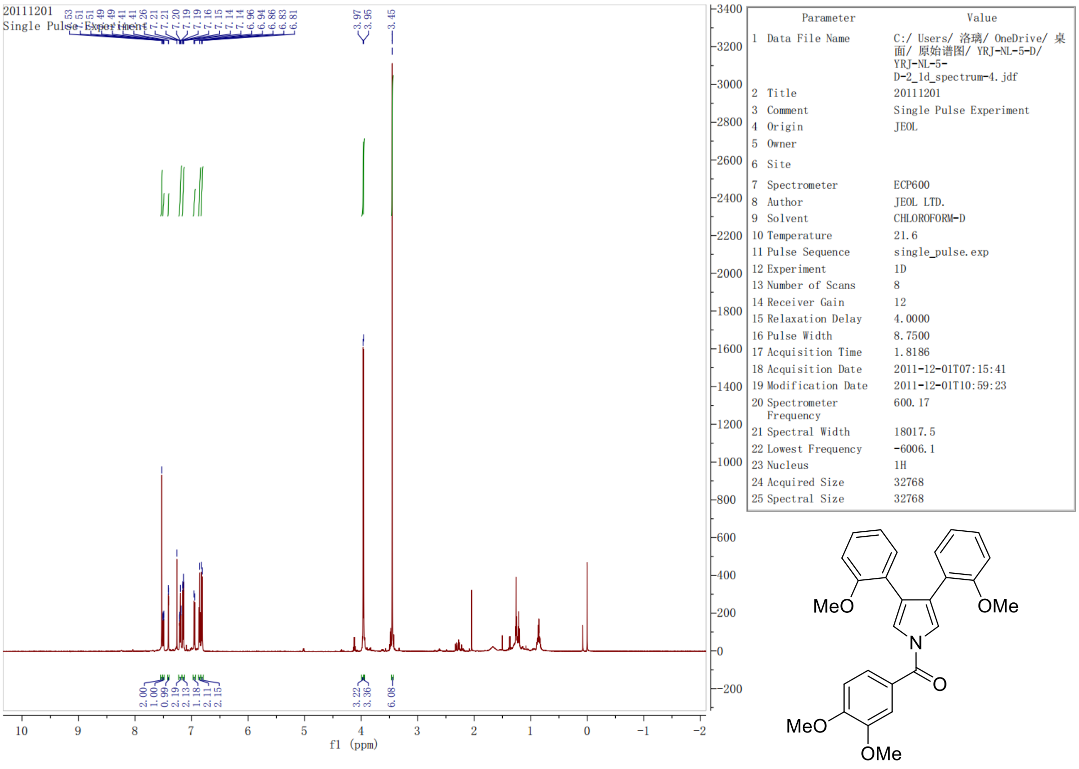

Figure S40. ^{1}H NMR spectrum of **1d**

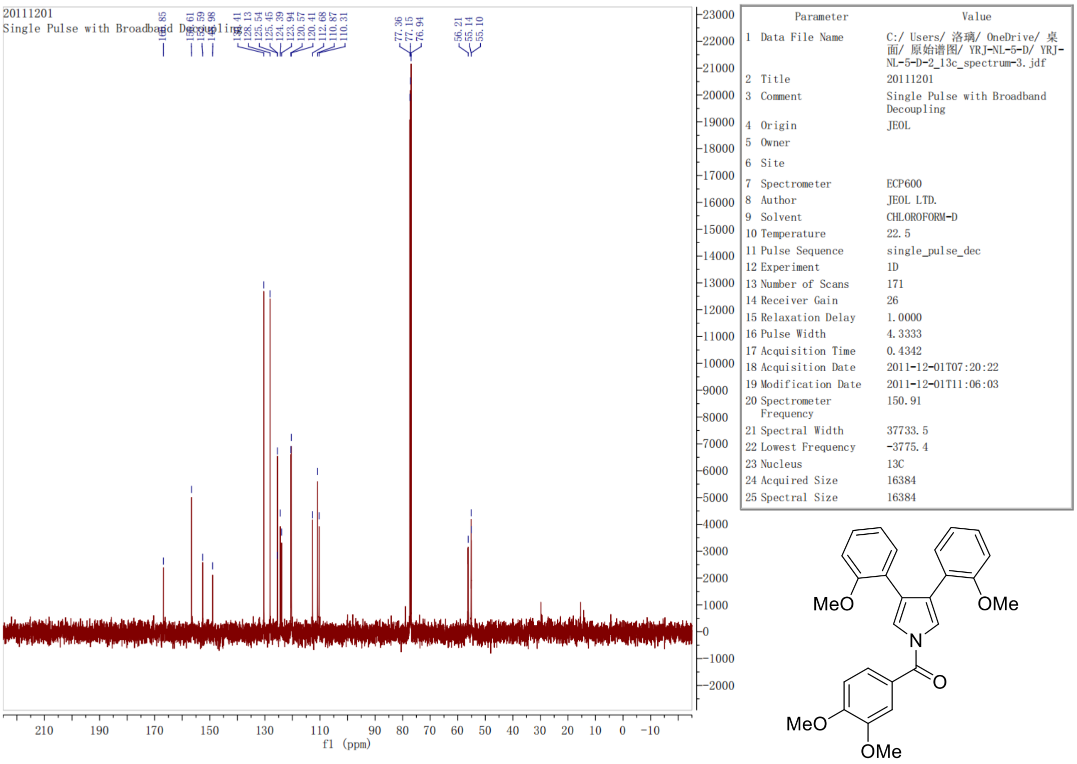

Figure S41. ^{13}C NMR spectrum of **1d**

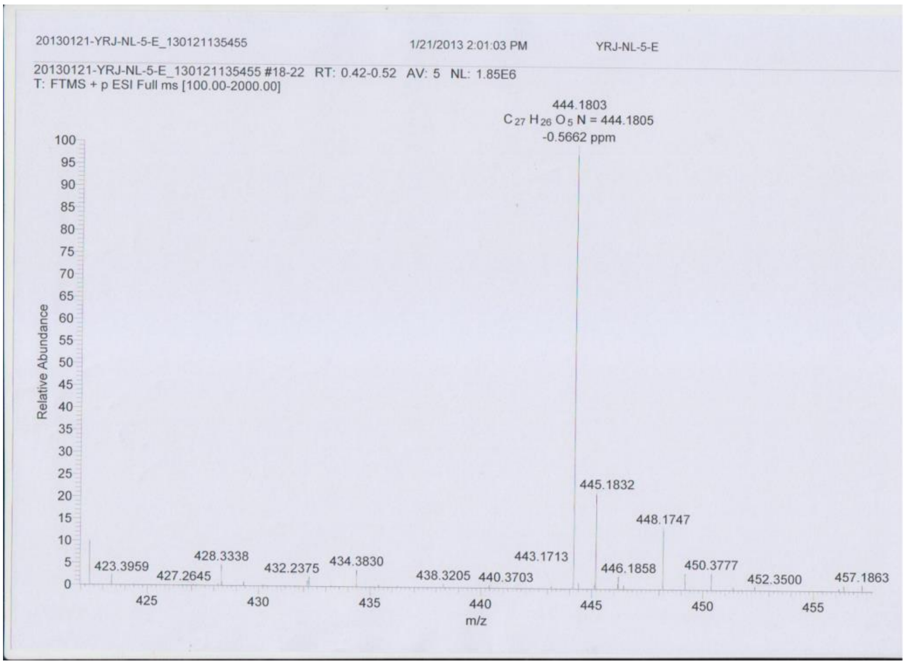

Figure S42. HRMS spectrum of **1e**

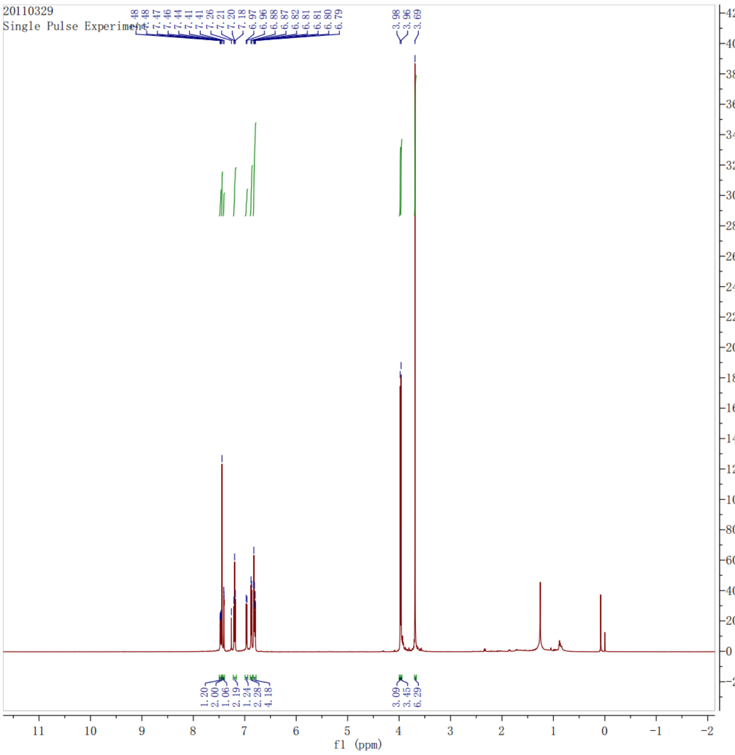

Parameter	Value
1 Data File Name	C:/ Users/ 洛璃/ OneDrive/ 桌面/ 原始谱图/ YRJ-NL-5-E/ YRJ-NL-5-E_1d_spectrum-3. jdf
2 Title	20110329
3 Comment	Single Pulse Experiment
4 Origin	JEOL
5 Owner	
6 Site	
7 Spectrometer	ECP600
8 Author	JEOL LTD.
9 Solvent	CHLOROFORM-D
10 Temperature	22.1
11 Pulse Sequence	single_pulse.exp
12 Experiment	1D
13 Number of Scans	8
14 Receiver Gain	13
15 Relaxation Delay	4.0000
16 Pulse Width	8.7500
17 Acquisition Time	1.8186
18 Acquisition Date	2011-03-29T13:44:02
19 Modification Date	2011-04-06T13:54:18
20 Spectrometer Frequency	600.17
21 Spectral Width	18017.5
22 Lowest Frequency	-6005.5
23 Nucleus	1H
24 Acquired Size	32768
25 Spectral Size	32768

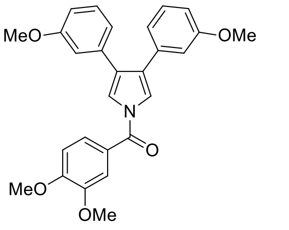

Figure S43. ^{1}H NMR spectrum of **1e**

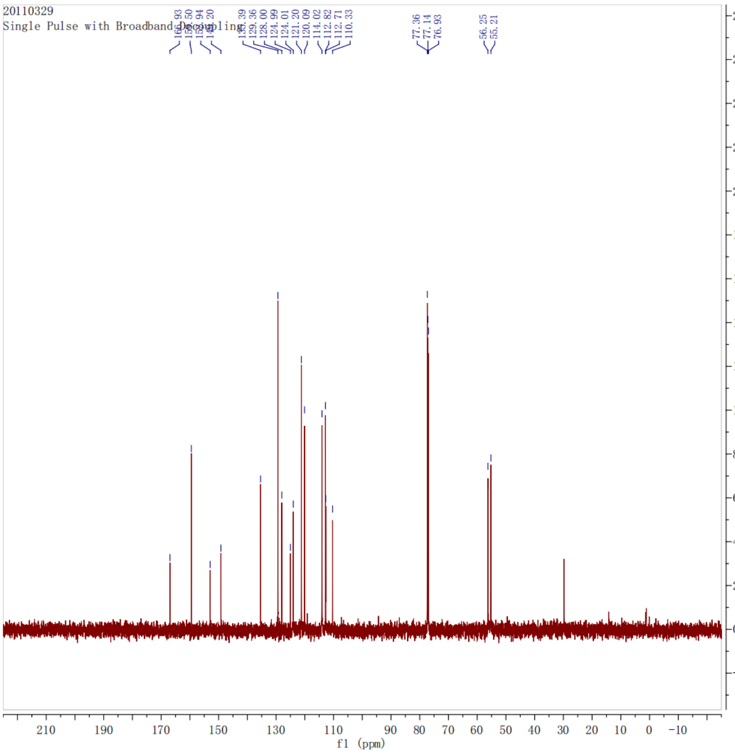

Parameter	Value
1 Data File Name	C:/ Users/ 洛璃/ OneDrive/ 桌面/ 原始谱图/ YRJ-NL-5-E/ YRJ-NL-5-E_13c_spectrum-3. jdf
2 Title	20110329
3 Comment	Single Pulse with Broadband Decoupling
4 Origin	JEOL
5 Owner	
6 Site	
7 Spectrometer	ECP600
8 Author	JEOL LTD.
9 Solvent	CHLOROFORM-D
10 Temperature	23.1
11 Pulse Sequence	single_pulse_dec
12 Experiment	1D
13 Number of Scans	158
14 Receiver Gain	26
15 Relaxation Delay	1.0000
16 Pulse Width	4.3333
17 Acquisition Time	0.4342
18 Acquisition Date	2011-03-29T13:48:24
19 Modification Date	2011-04-06T14:32:05
20 Spectrometer Frequency	150.91
21 Spectral Width	37733.5
22 Lowest Frequency	-3775.4
23 Nucleus	13C
24 Acquired Size	16384
25 Spectral Size	16384

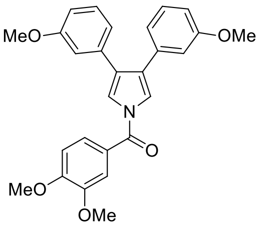

Figure S44. ^{13}C NMR spectrum of **1e**

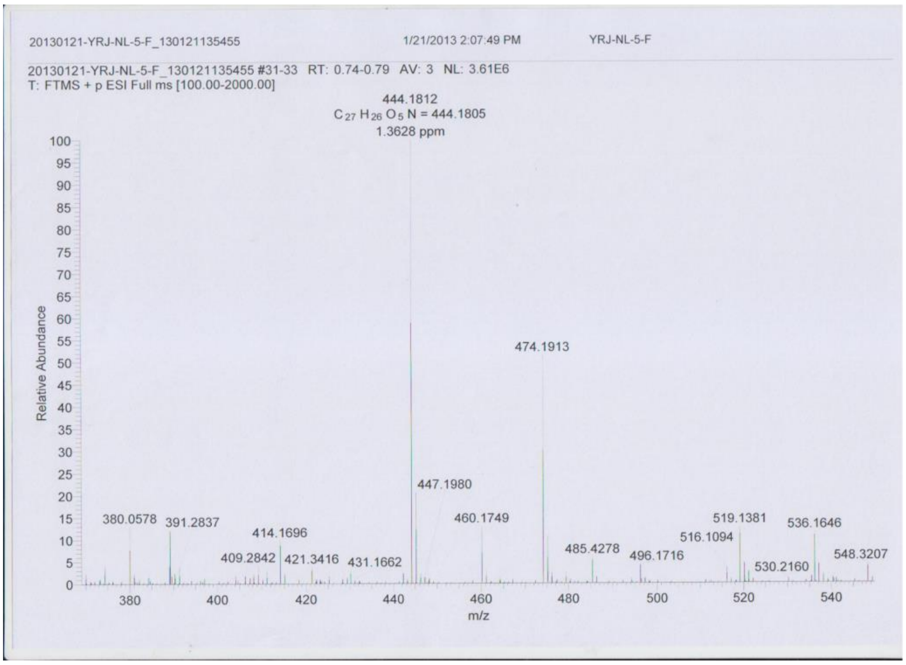

Figure S45. HRMS spectrum of **1f**

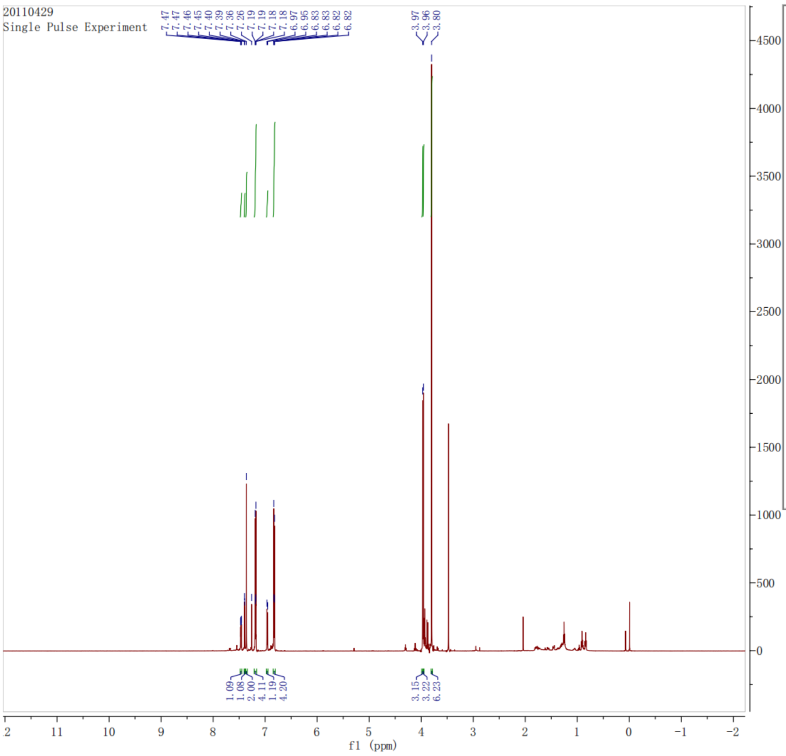

Parameter	Value
1 Data File Name	C:/ Users/ 洛璃/ OneDrive/ 桌面/ 原始谱图/ YRJ-NL-5-F/ YRJ-NL-5-F_1d_spectrum-3. jdf
2 Title	20110429
3 Comment	Single Pulse Experiment
4 Origin	JEOL
5 Owner	
6 Site	
7 Spectrometer	ECP600
8 Author	JEOL LTD.
9 Solvent	CHLOROFORM-D
10 Temperature	21.2
11 Pulse Sequence	single_pulse.exp
12 Experiment	1D
13 Number of Scans	8
14 Receiver Gain	14
15 Relaxation Delay	4.0000
16 Pulse Width	8.7500
17 Acquisition Time	1.8186
18 Acquisition Date	2011-04-29T14:18:32
19 Modification Date	2011-05-05T10:29:17
20 Spectrometer Frequency	600.17
21 Spectral Width	18017.5
22 Lowest Frequency	-6006.7
23 Nucleus	1H
24 Acquired Size	32768
25 Spectral Size	32768

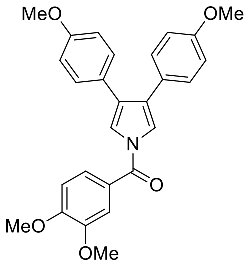

Figure S46. ^{1}H NMR spectrum of **1f**

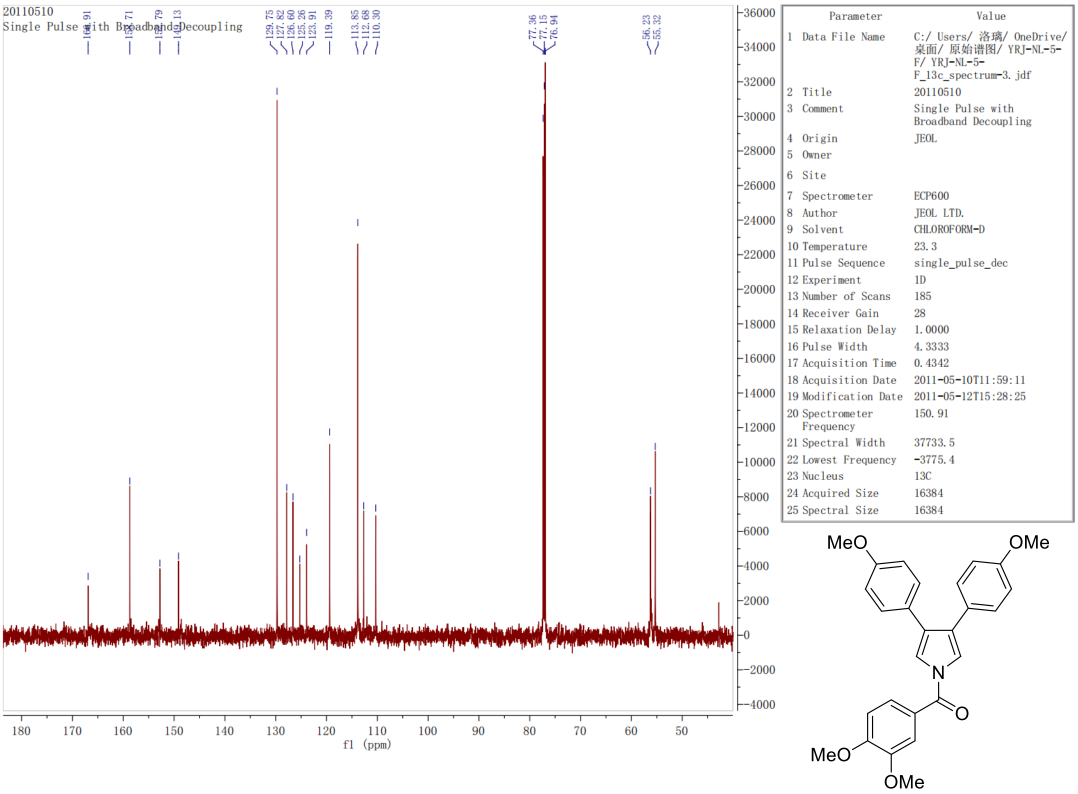

Figure S47. ^{13}C NMR spectrum of **1f**

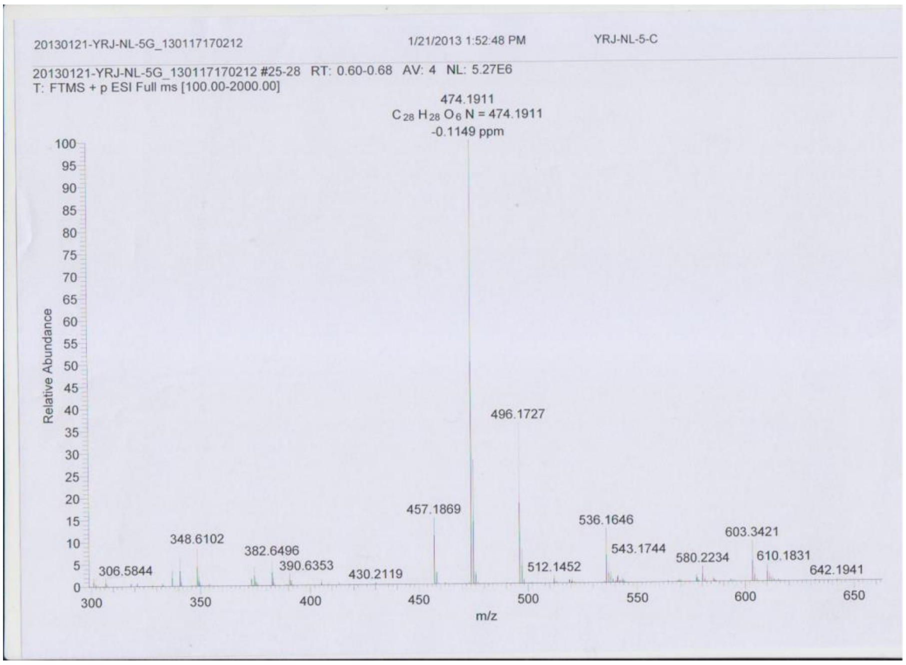

Figure S48. HRMS spectrum of **1g**

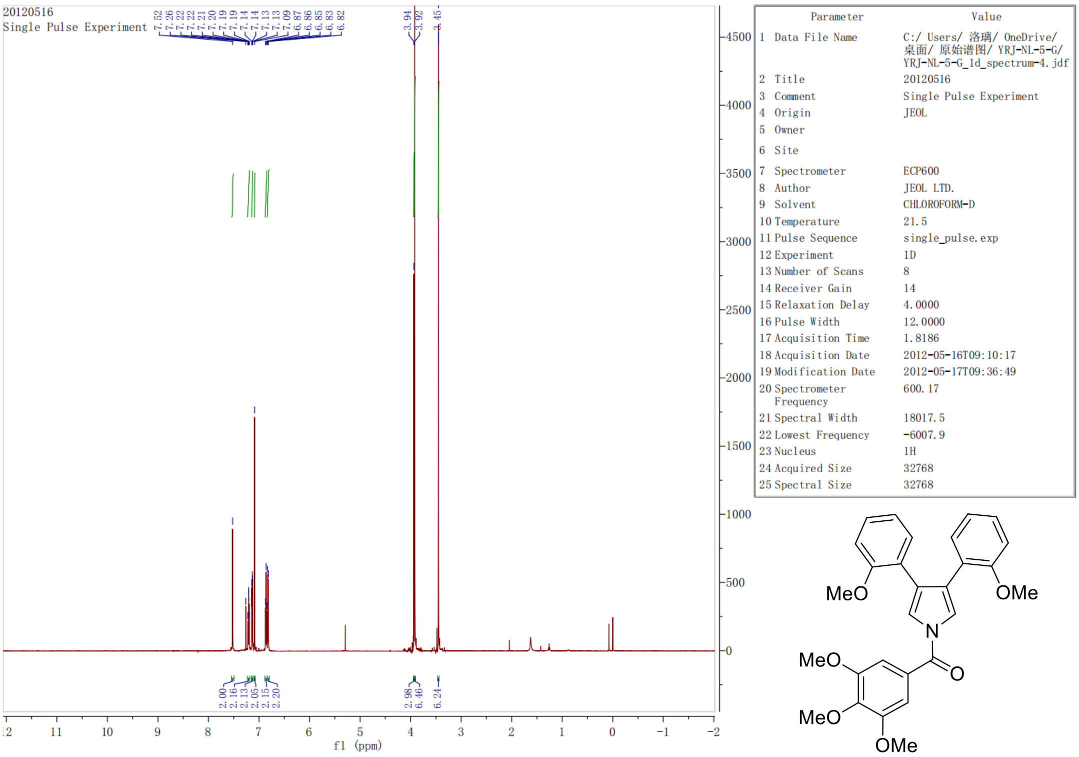

Figure S49. ^{1}H NMR spectrum of **1g**

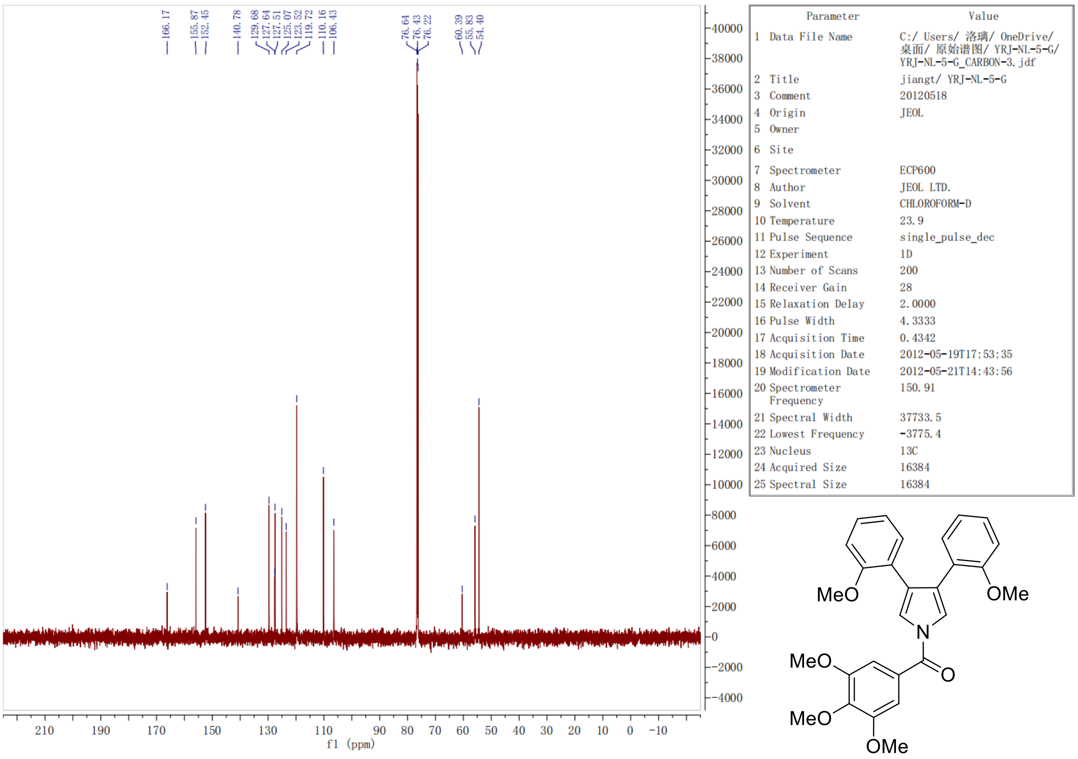

Figure S50. ^{13}C NMR spectrum of **1g**

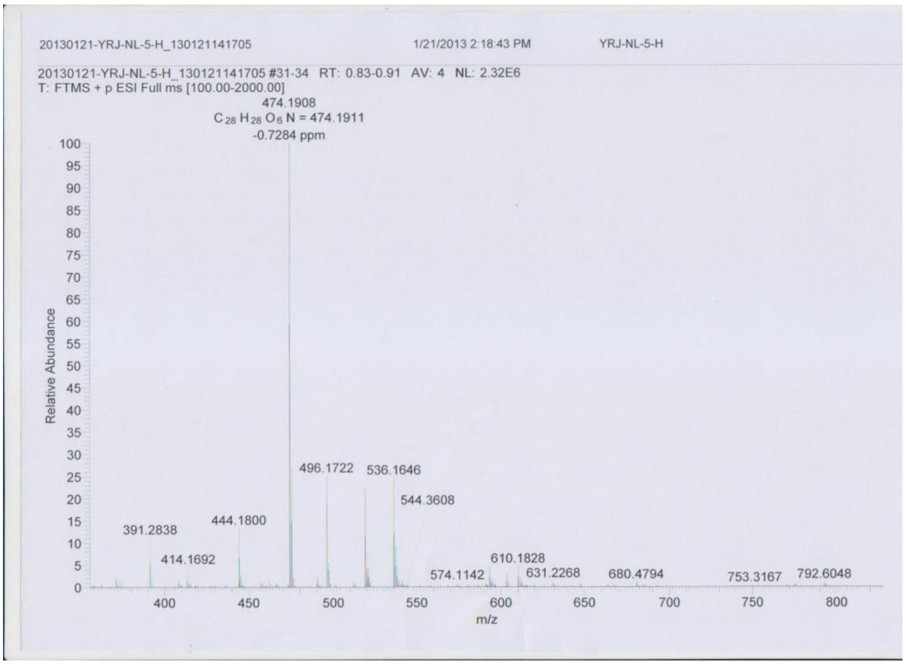

Figure S51. HRMS spectrum of **1h**

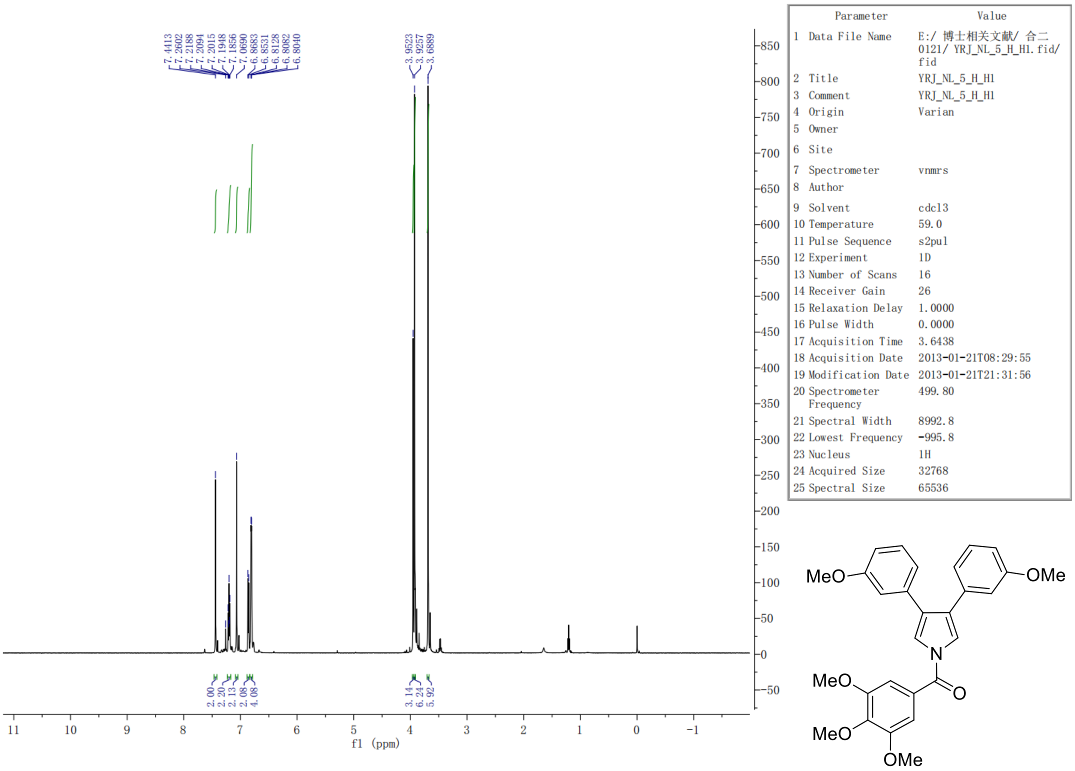

Figure S52. ^{1}H NMR spectrum of **1h**

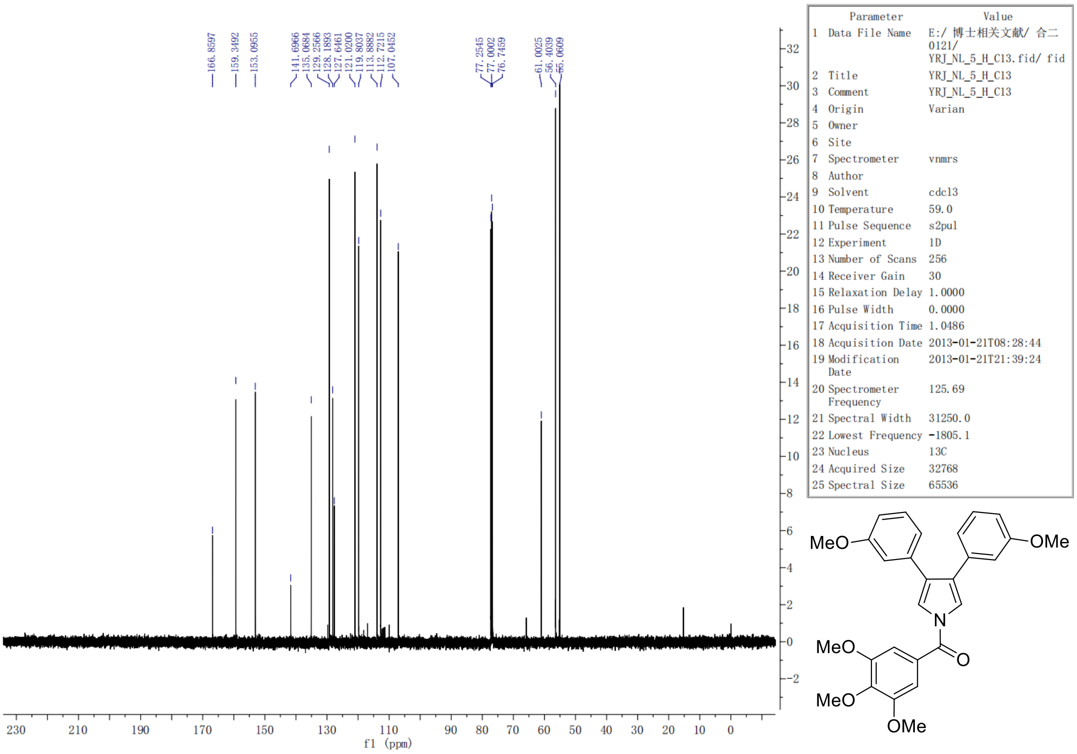

Figure S53. ^{13}C NMR spectrum of **1h**

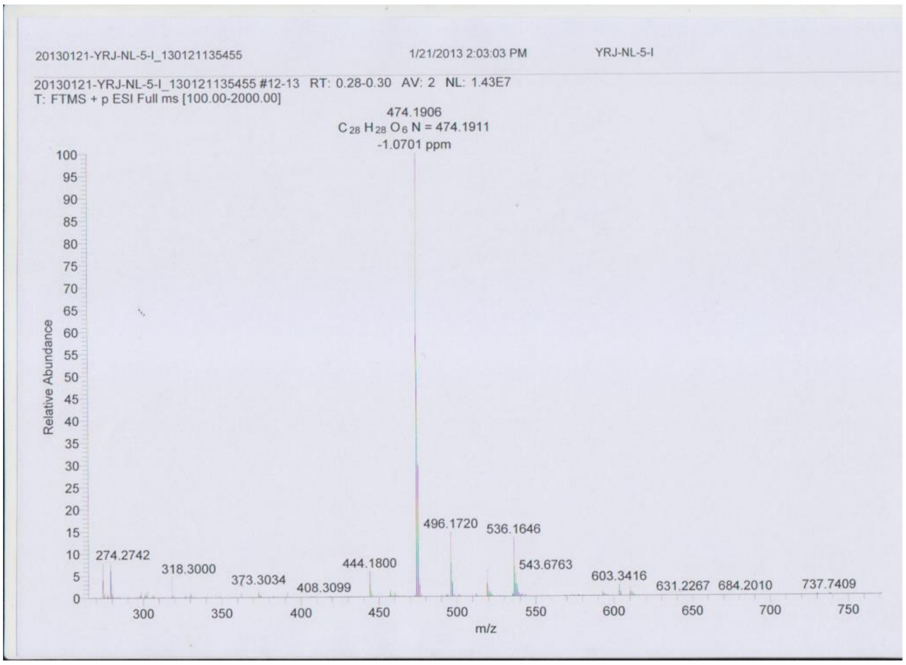

Figure S54. HRMS spectrum of **1i**

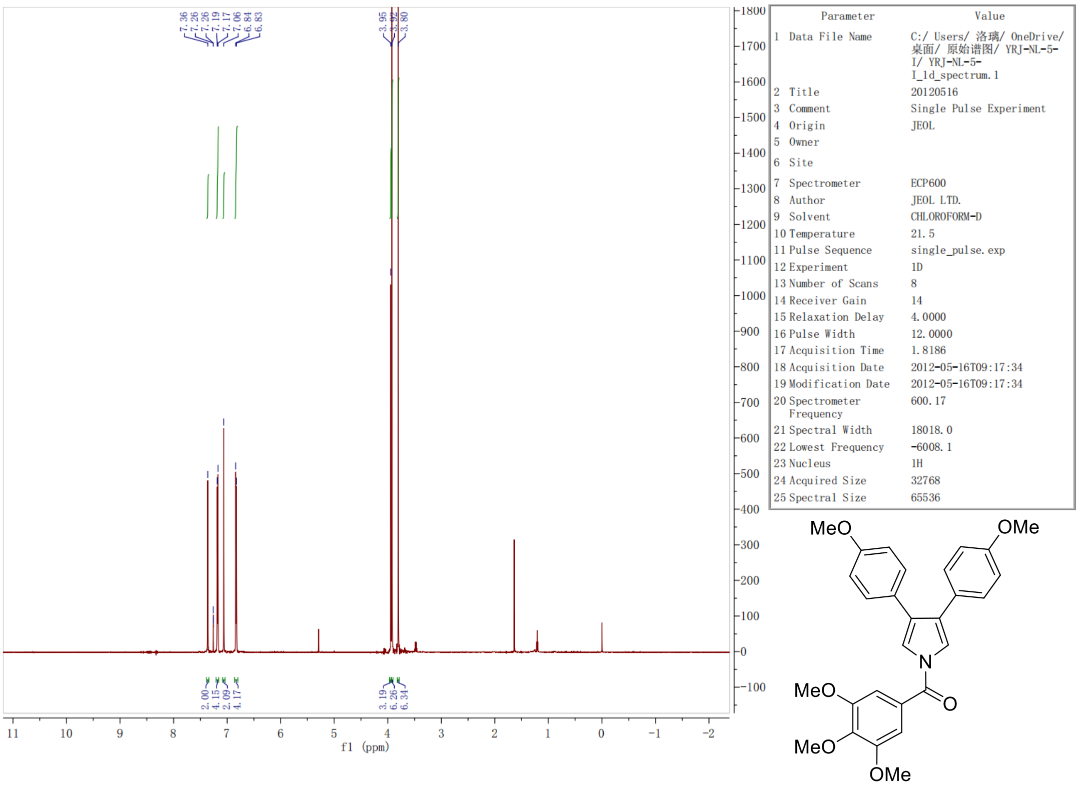

Figure S55. ^{1}H NMR spectrum of **1i**

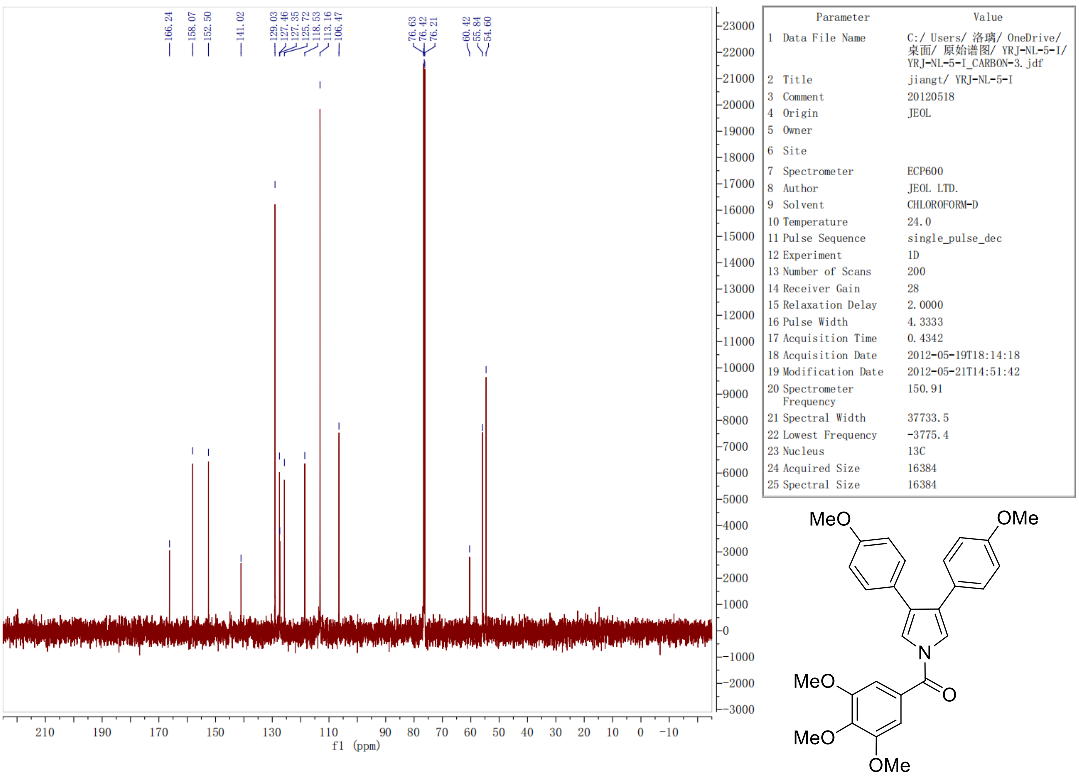

Figure S56. ^{13}C NMR spectrum of **1i**

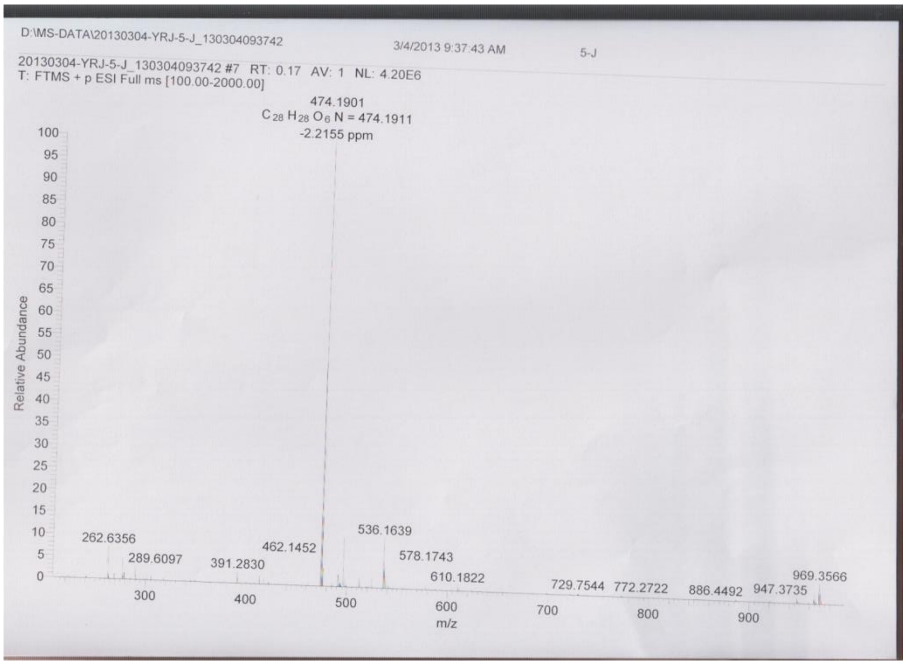

Figure S57. HRMS spectrum of **1j**

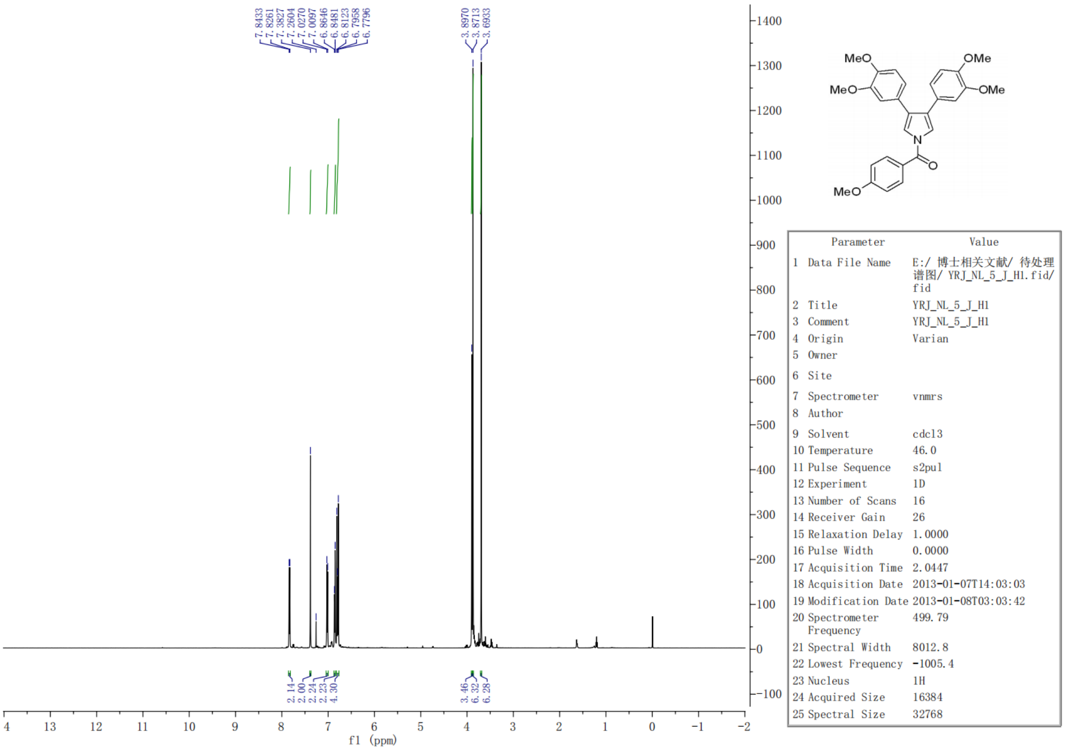

Figure S58. ^{1}H NMR spectrum of **1j**

YRJ-NL-5-J

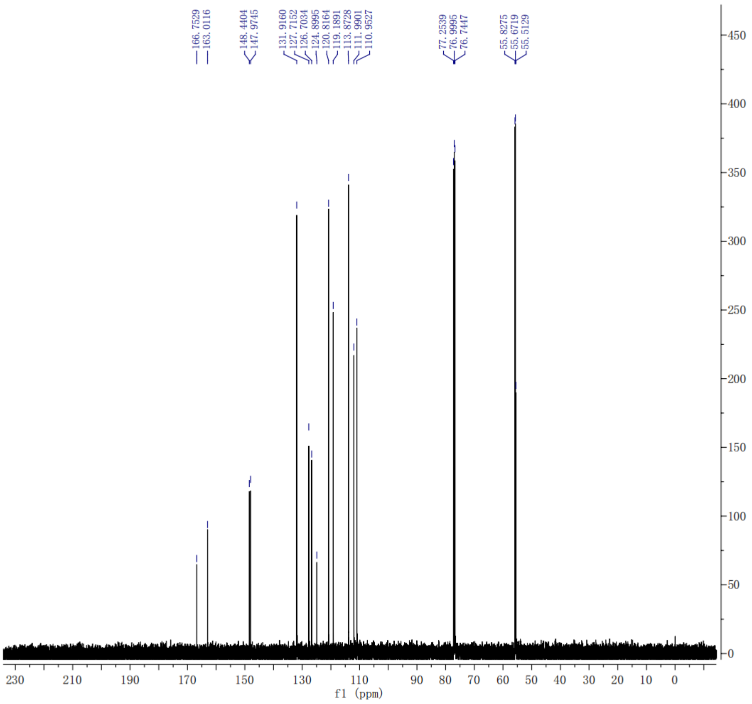

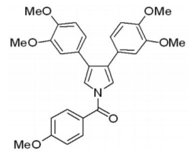

	Parameter	Value
1	Data File Name	E:/ 博士相关文献/ 待处理谱图/ YRJ_NL_5_J_C13.fid/fid
2	Title	YRJ_NL_5_J_C13
3	Comment	YRJ_NL_5_J_C13
4	Origin	Varian
5	Owner	
6	Site	
7	Spectrometer	vnmrs
8	Author	
9	Solvent	cdcl3
10	Temperature	46.0
11	Pulse Sequence	s2pul
12	Experiment	1D
13	Number of Scans	128
14	Receiver Gain	60
15	Relaxation Delay	1.0000
16	Pulse Width	0.0000
17	Acquisition Time	1.0486
18	Acquisition Date	2013-01-07T14:04:12
19	Modification Date	2013-01-08T03:09:06
20	Spectrometer Frequency	125.69
21	Spectral Width	31250.0
22	Lowest Frequency	-1805.2
23	Nucleus	13C
24	Acquired Size	32768
25	Spectral Size	65536

Figure S59. ^{13}C NMR spectrum of **1j**

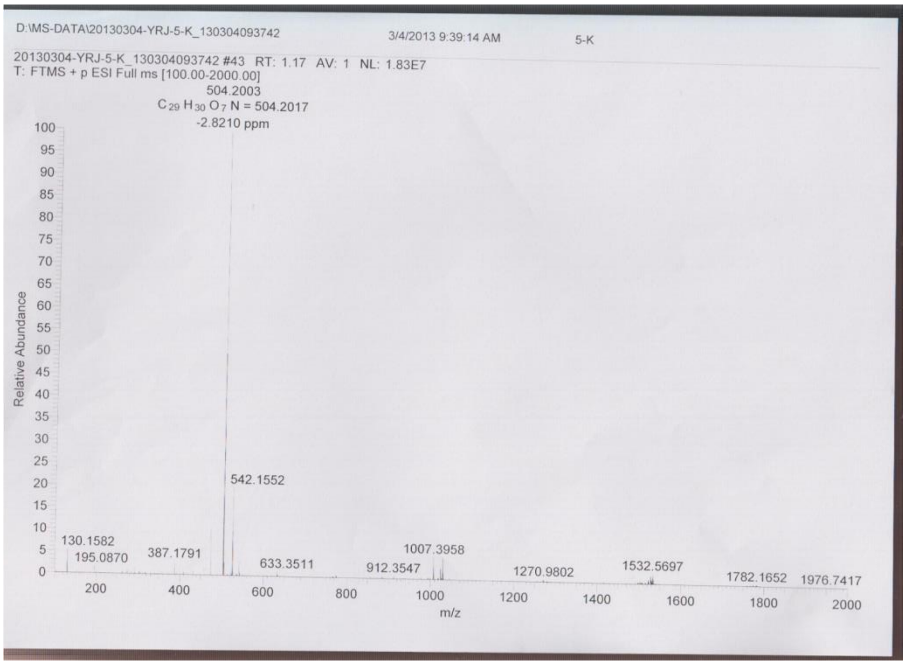

Figure S60. HRMS spectrum of **1k**

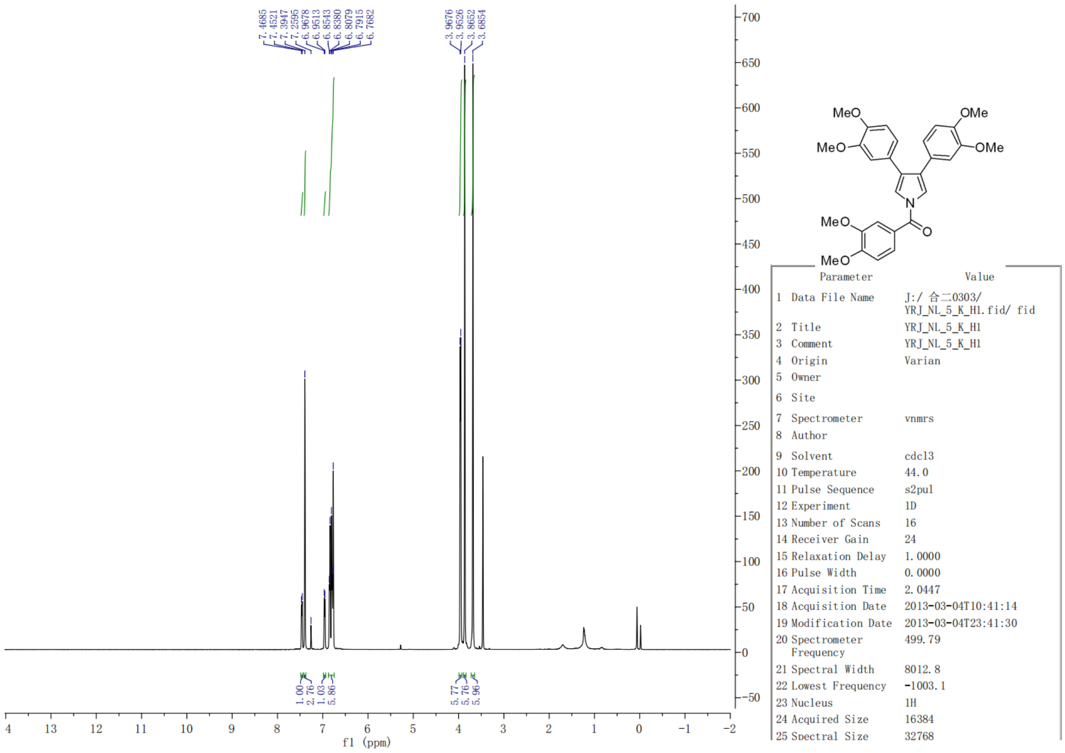

Figure S61. ^{1}H NMR spectrum of **1k**

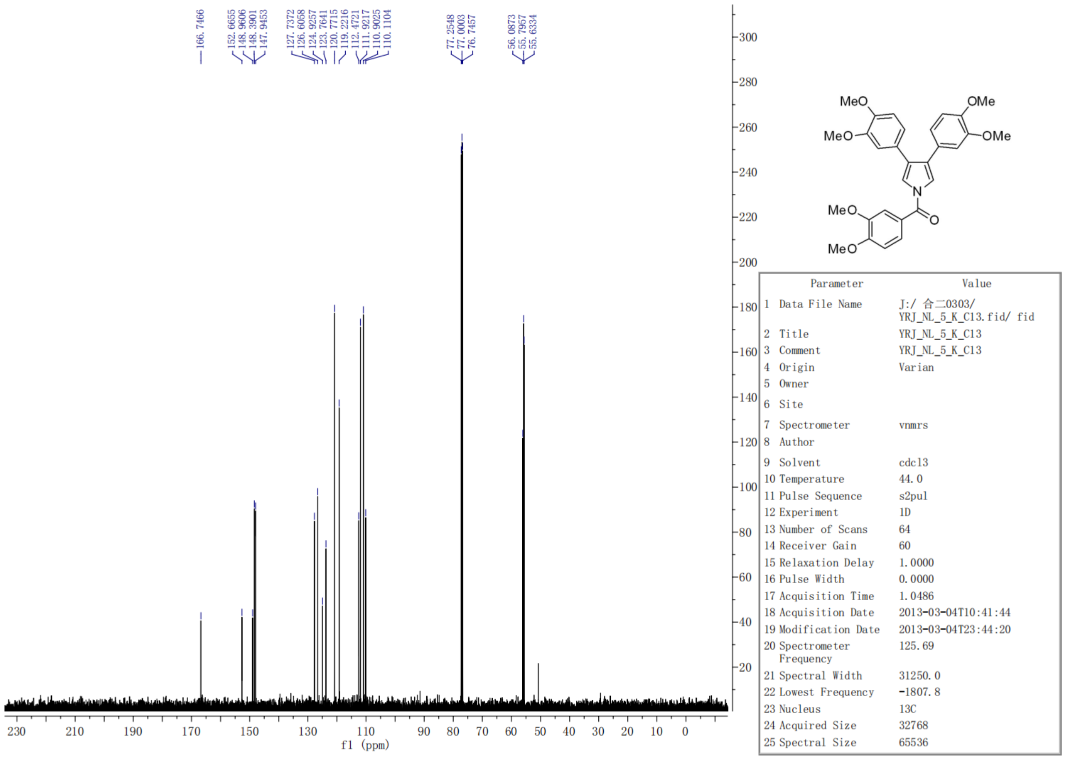

Figure S62. ^{13}C NMR spectrum of **1k**

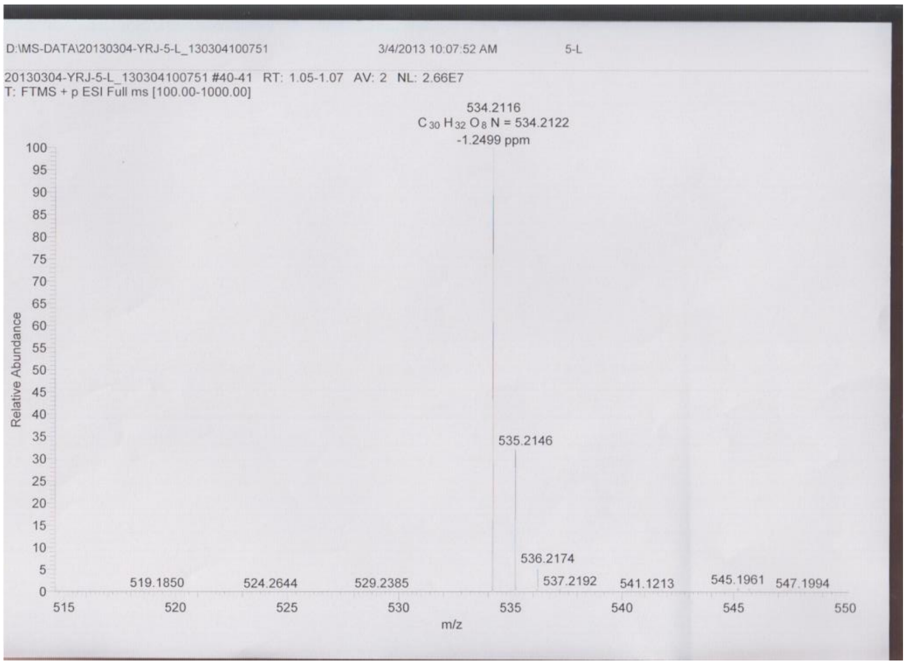

Figure S63. HRMS spectrum of **1l**

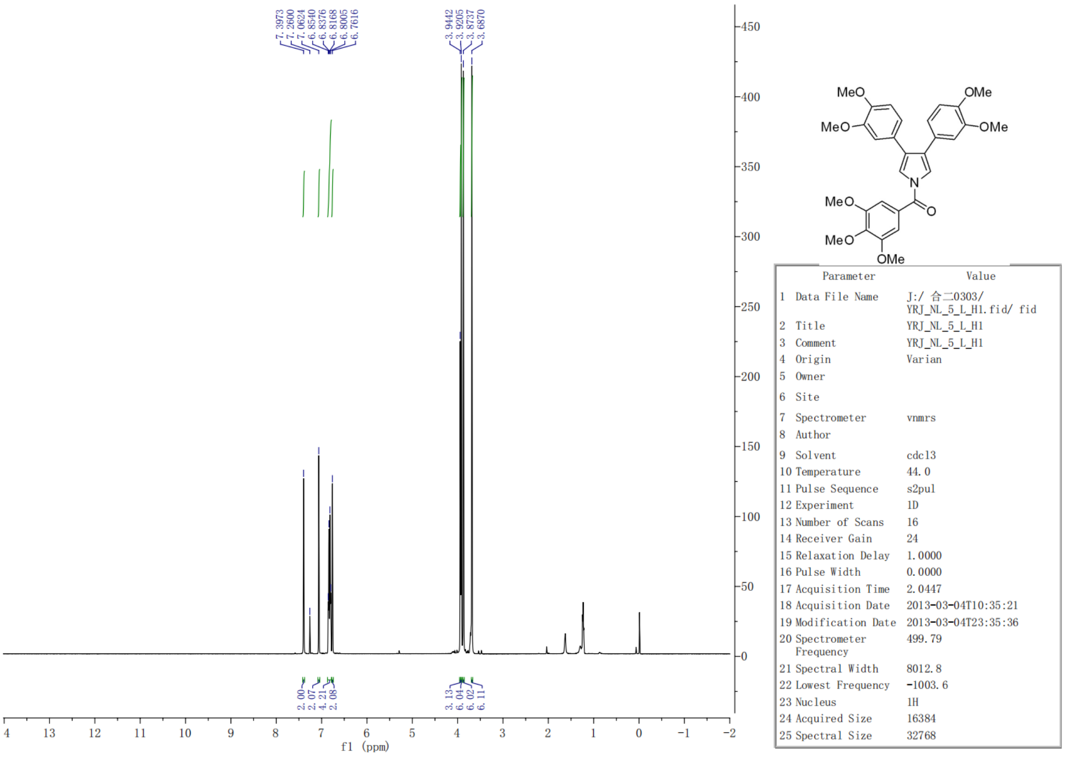

Figure S64. ^{1}H NMR spectrum of **1l**

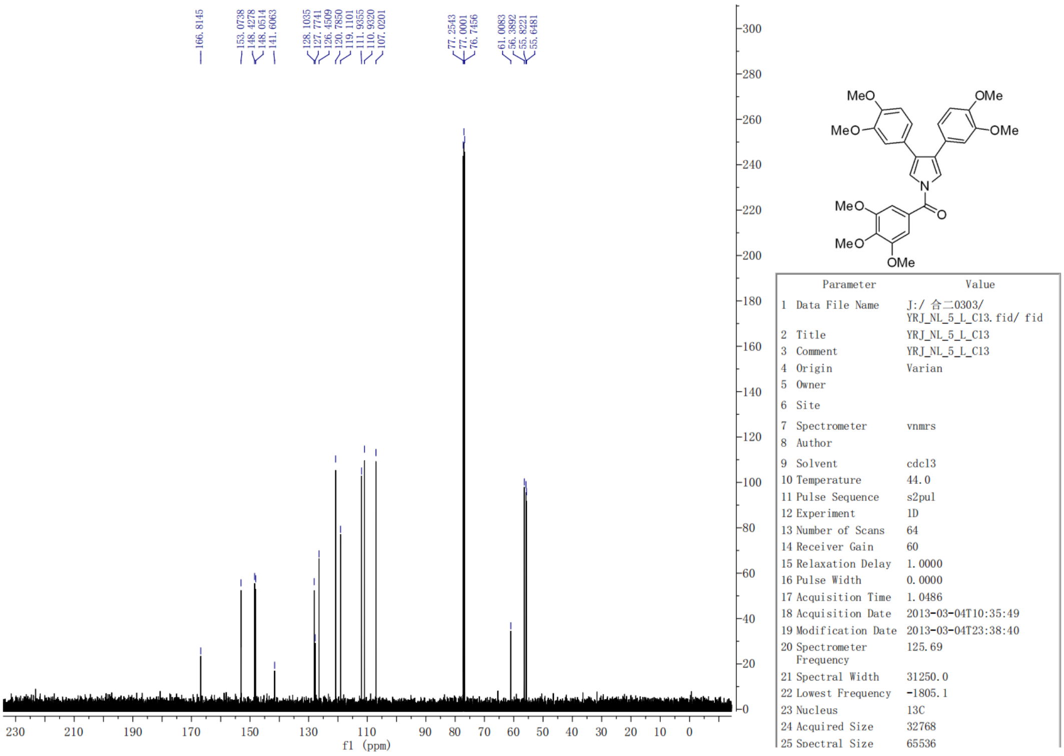

Figure S65. ^{13}C NMR spectrum of **1l**

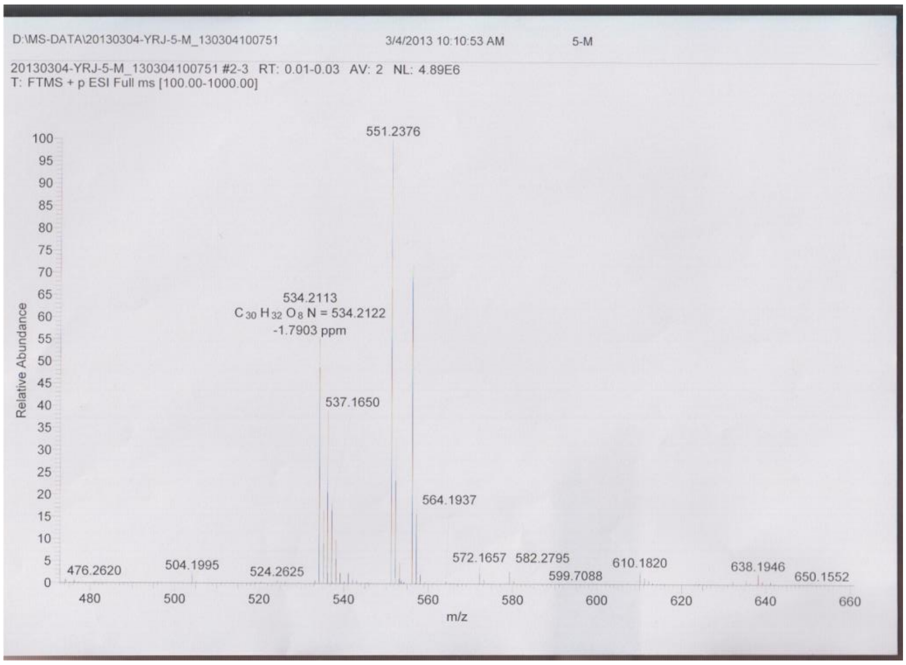

Figure S66. HRMS spectrum of **1m**

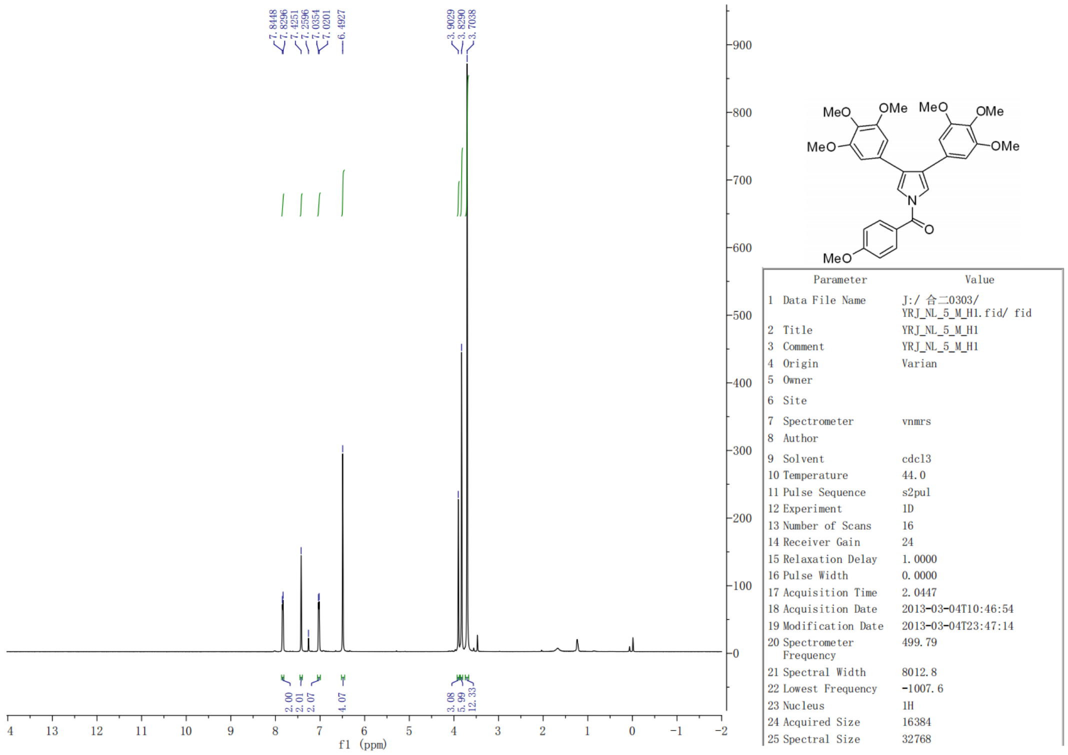

Figure S67. ^{1}H NMR spectrum of **1m**

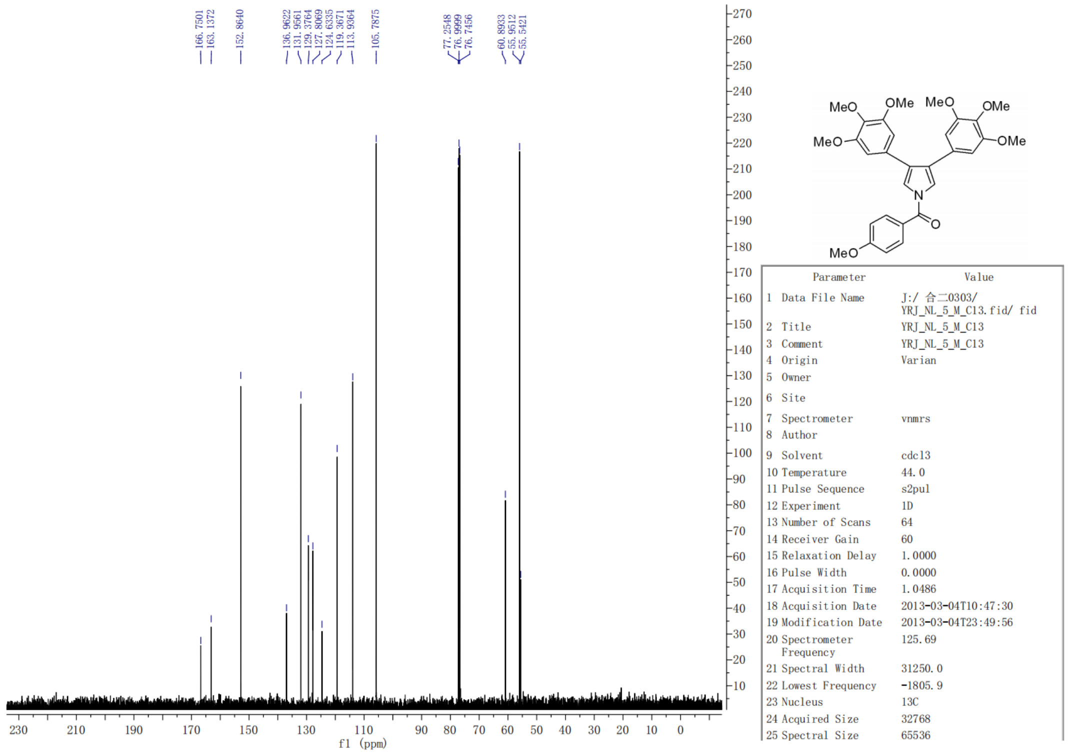

Figure S68. ^{13}C NMR spectrum of **1m**

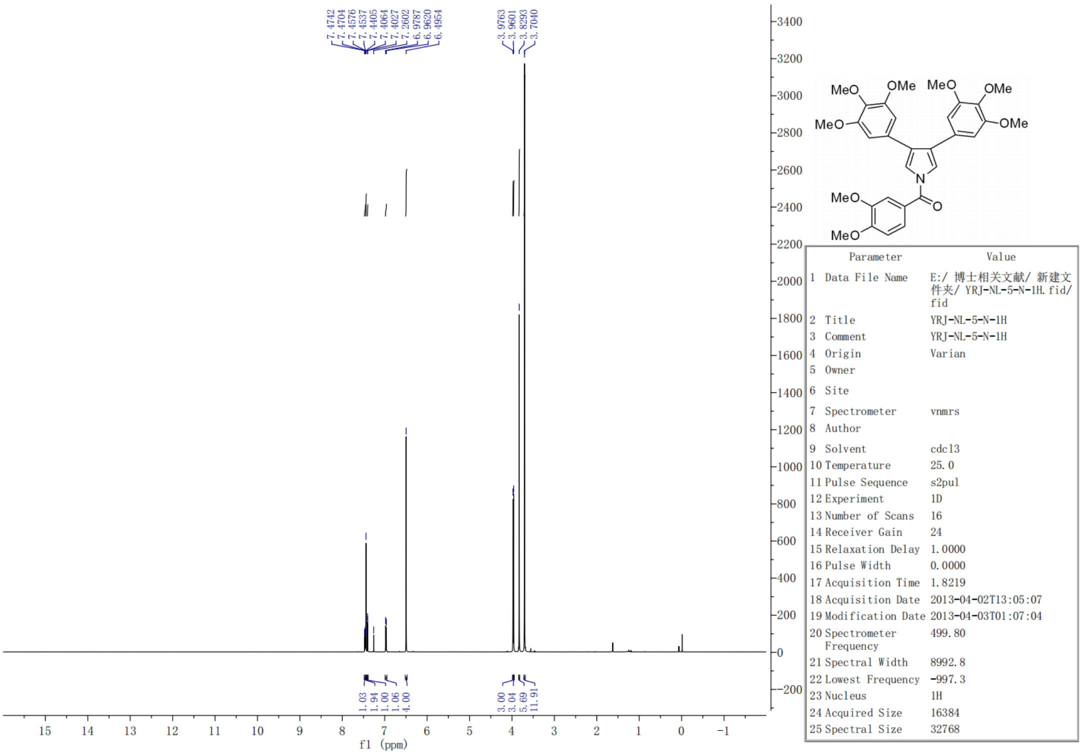

Figure S69. ^{1}H NMR spectrum of **1n**

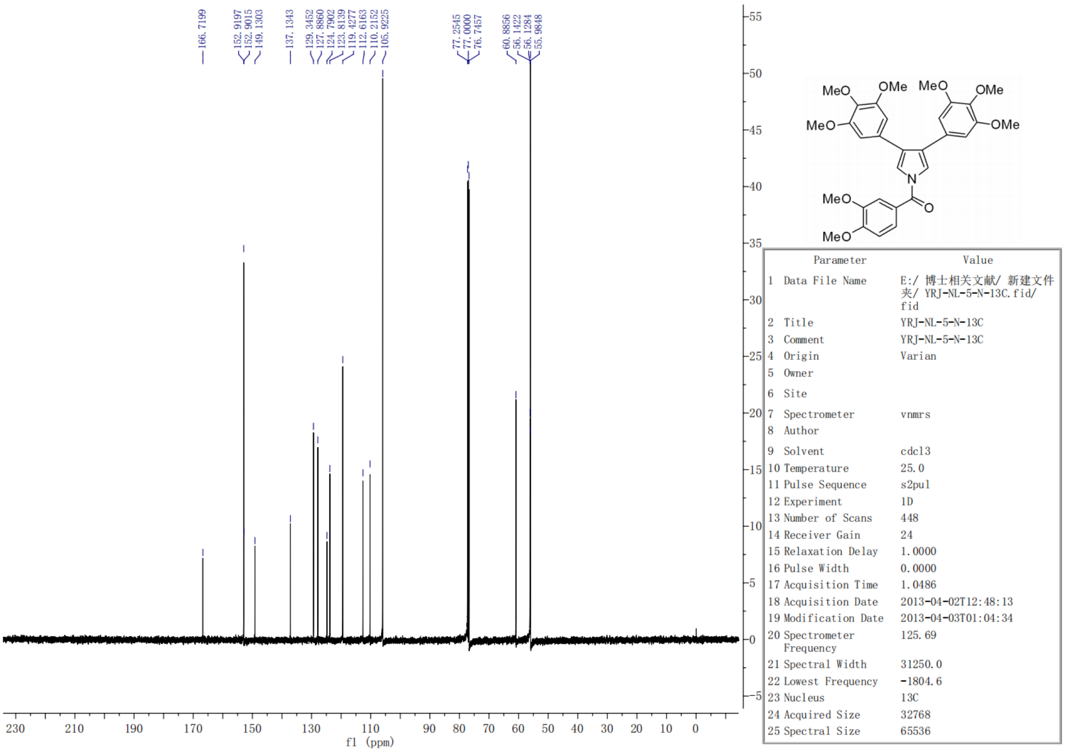

Figure S70. ^{13}C NMR spectrum of **1n**

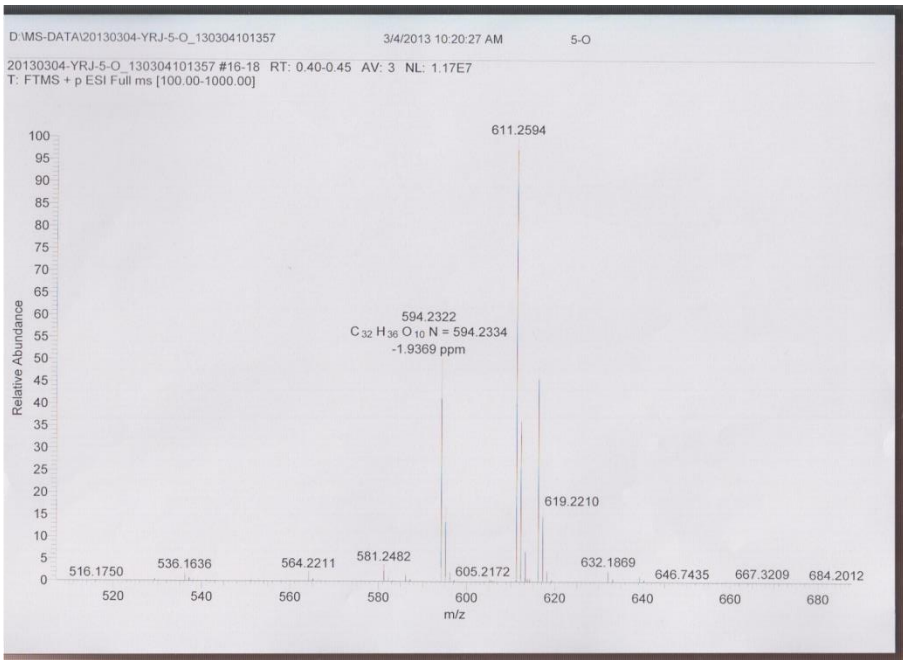

Figure S71. HRMS spectrum of **1o**

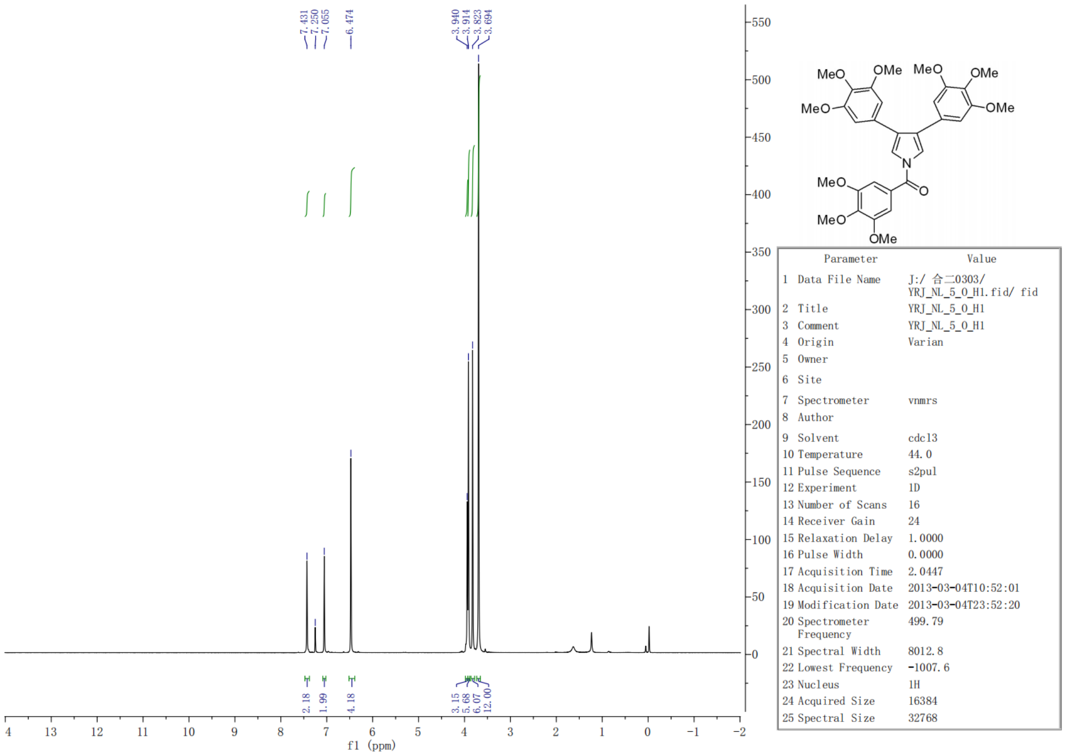

Figure S72. ^{1}H NMR spectrum of **1o**

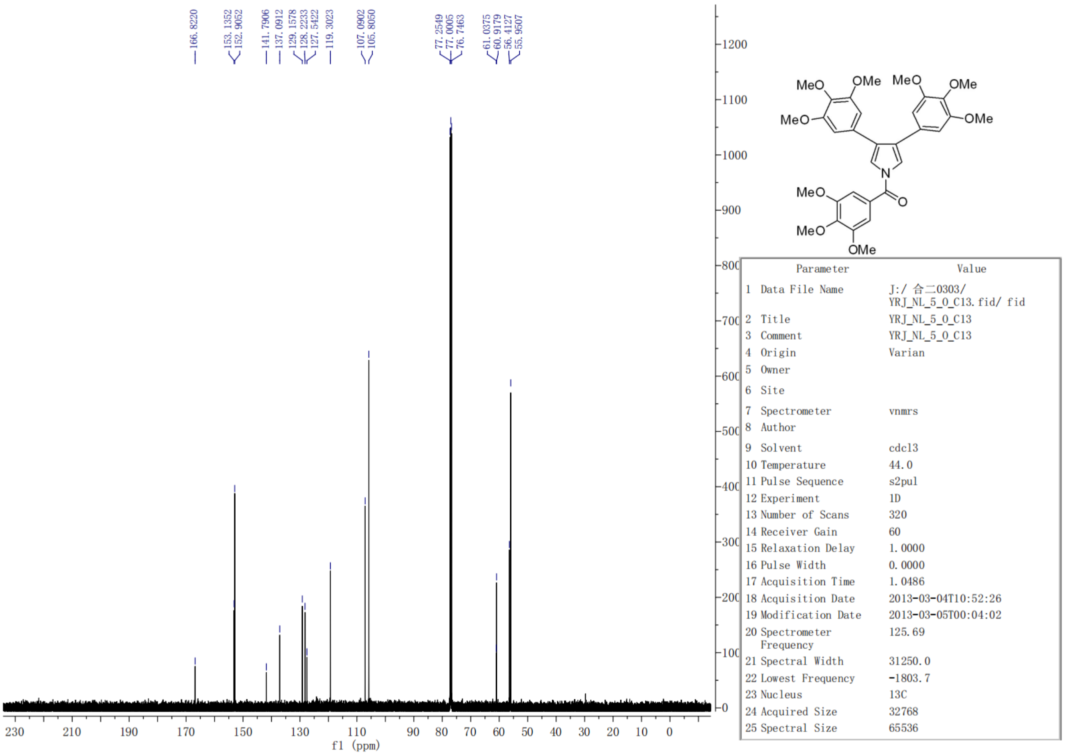

Figure S73. ^{13}C NMR spectrum of **1o**

Table S1 The cytotoxicity of the permethylated Neolamellarin A derivatives **1a - 1o** at gradient concentrations on PC12 cell line

Compound	Concentration (μM)	Cell viability ± SD (100%) [a]
**Blank control**		100
**1a**	2.5	109.55 ± 2.57
	5	114.88 ± 2.40
	10	126.65 ± 2.75
	20	143.33 ± 1.81
**1b**	2.5	112.50 ± 3.35
	5	120.05 ± 2.51
	10	131.64 ± 1.91
	20	142.52 ± 3.84
**1c**	2.5	117.50 ± 2.18
	5	126.36 ± 2.63
	10	144.89 ± 5.27
	20	162.47 ± 5.41
**1d**	2.5	110.87 ± 6.78
	5	113.82 ± 1.92
	10	112.02 ± 3.75
	20	109.71 ± 3.56
**1e**	2.5	111.54 ± 1.93
	5	112.52 ± 2.73
	10	114.40 ± 4.32
	20	116.44 ± 5.44
**1f**	2.5	105.19 ± 0.41
	5	118.95 ± 6.60
	10	126.46 ± 3.66
	20	128.42 ± 6.00
**1g**	2.5	106.50 ± 2.14
	5	105.91 ± 3.74
	10	109.93 ± 2.07
	20	114.22 ± 3.37
**1h**	2.5	104.35 ± 2.67
	5	111.95 ± 4.52

Compound	Concentration (μM)	Cell viability ± SD (100%) [a]
	10	122.42 ± 4.50
	20	119.64 ± 2.05
**1i**	2.5	120.04 ± 4.76
	5	125.62 ± 4.35
	10	126.80 ± 3.13
	20	114.50 ± 2.73
**1j**	2.5	104.55 ± 1.85
	5	105.61 ± 1.26
	10	113.76 ± 2.14
	20	104.89 ± 3.92
**1k**	2.5	106.24 ± 1.52
	5	104.61 ± 0.62
	10	100.21 ± 2.89
	20	95.64 ± 2.10
**1l**	2.5	110.38 ± 2.02
	5	107.82 ± 3.05
	10	116.44 ± 1.52
	20	115.45 ± 4.72
**1m**	2.5	106.37 ± 3.19
	5	104.45±3.68
	10	104.40±4.30
	20	90.56 ± 3.00
**1n**	2.5	103.97 ± 3.32
	5	101.61 ± 3.04
	10	107.02 ± 3.76
	20	97.37 ± 3.06
**1o**	2.5	109.02 ± 3.18
	5	106.50 ± 3.41
	10	108.77 ± 0.49
	20	100.00 ± 3.75

[a] Each value was calculated from three independent experiments. The data were shown as mean ± SD deviation.

Table S2 The neuroprotective activity of the permethylated Neolamellarin A derivatives **1a - 1o** at gradient concentrations on PC12 cell death induced by glutamate

Compound	Concentration (μM)	Cell viability ± SD (100%) [a]
**Blank control**		100
**Negative control (glutamate)**	8	35.92 ± 3.01
**HupA (positive control)**	100	45.55 ± 5.77
**1a**	2.5	43.89 ± 4.17
	5	45.24 ± 2.88
	10	50.75 ± 4.38
	20	51.61 ± 3.23
**1b**	2.5	42.68 ± 3.71
	5	44.48 ± 6.37
	10	52.20 ± 7.03
	20	61.68 ± 8.32
**1c**	2.5	45.00 ± 3.53
	5	56.69 ± 7.06
	10	85.01 ± 5.22
	20	99.73 ± 10.49
**1d**	2.5	50.97 ± 3.33
	5	50.63 ± 4.07
	10	67.54 ± 5.60
	20	79.27 ± 2.41
**1e**	2.5	45.51 ± 4.72
	5	52.75 ± 2.89
	10	57.28 ± 7.00
	20	73.87 ± 3.14
**1f**	2.5	39.95 ± 1.59
	5	48.00 ± 2.39
	10	59.63 ± 2.53
	20	101.56 ± 3.79
**1g**	2.5	46.56 ± 2.42
	5	40.99 ± 3.16
	10	53.40 ± 3.90
	20	55.35 ± 1.86

Compound	Concentration (μM)	Cell viability ± SD (100%) [a]
**1h**	2.5	41.51 ± 1.37
	5	51.51 ± 5.10
	10	79.41 ± 3.13
	20	119.20 ± 4.86
**1i**	2.5	46.35 ± 3.19
	5	57.84 ± 3.70
	10	80.34 ± 6.26
	20	71.75 ± 5.67
**1j**	2.5	44.40 ± 5.99
	5	36.06 ± 3.52
	10	43.68 ± 4.41
	20	67.64 ± 6.34
**1k**	2.5	38.24 ± 3.06
	5	45.06 ± 1.49
	10	67.26 ± 2.20
	20	84.79 ± 5.62
**1l**	2.5	53.15 ± 3.28
	5	43.38 ± 2.35
	10	47.41 ± 4.39
	20	49.26 ± 5.43
**1m**	2.5	38.38 ± 1.75
	5	50.91 ± 3.36
	10	86.42 ± 5.66
	20	94.21 ± 4.07
**1n**	2.5	48.27 ± 4.11
	5	65.74 ± 2.95
	10	102.38 ± 7.61
	20	106.31 ± 3.22
**1o**	2.5	47.09 ± 5.51
	5	50.18 ± 0.79
	10	70.22 ± 6.79
	20	113.90 ± 10.11

[a] Each value was calculated from three independent experiments. The data were shown as mean ± SD deviation.
